# The second messenger c-di-AMP controls natural competence via ComFB signaling protein

**DOI:** 10.1038/s41421-025-00816-x

**Published:** 2025-08-01

**Authors:** Sherihan Samir, Sofía Doello, Andreas M. Enkerlin, Erik Zimmer, Michael Haffner, Teresa Müller, Lisa Dengler, Stilianos P. Lambidis, Shamphavi Sivabalasarma, Sonja-Verena Albers, Khaled A. Selim

**Affiliations:** 1 Interfaculty Institute of Microbiology and Infection Medicine, Cluster of Excellence “Controlling Microbes to Fight Infections - CMFI”, Tübingen University, Tübingen, Germany; 2 Microbiology Department, Faculty of Science, Ain Shams University, Cairo, Egypt; 3 Microbial Biochemistry Group, Institute of Phototrophic Microbiology, Heinrich-Heine University Düsseldorf, Universitätsstraße 1, 40225 Düsseldorf, Germany; 4 Molecular Biology of Archaea, Freiburg University, Freiburg, Germany

**Keywords:** DNA metabolism, Stress signalling, Proteomics

Dear Editor,

Natural competence requires a contractile pilus system. Here, we provide evidence that the pilus biogenesis and natural competence in cyanobacteria are regulated by the second messenger c-di-AMP. Furthermore, we show that the ComFB signaling protein is a novel c-di-AMP-receptor protein, widespread in bacterial phyla, and required for pilus biogenesis and DNA uptake.

Cyclic di-AMP (c-di-AMP) is one of the recently discovered di-nucleotide-type second messengers^1^. In cyanobacteria, c-di-AMP controls diurnal metabolism via its binding to the carbon control protein SbtB to regulate glycogen metabolism^2^. Although important roles for c-di-AMP have been acknowledged since its discovery (e.g., in osmoregulation)^1–4^, recent studies suggested broader regulatory impacts of c-di-AMP signaling with further functions yet to be elucidated^3,4^ (see Supplementary Text). For instance, a role for c-di-AMP in controlling natural competence has been speculated^5^, although the molecular mechanism remained elusive^5^. In this study, we aimed to investigate the involvement of c-di-AMP in natural competence.

Natural competence involves a contractile pilus system and an assemblage of competence-accessory proteins^6,7^ (see Supplementary Text). To test the involvement of c-di-AMP in cyanobacterial natural competence, we compared the ability of the wild-type (WT) *Synechocystis* and the c-di-AMP-free Δ*dacA* mutant^2^ to take up DNA. The Δ*dacA* mutant showed significantly lower transformation efficiency than the WT, implying an essential role for c-di-AMP in natural competence (Fig. 1a; Supplementary Fig. S1). Complementing Δ*dacA* restored the transformation efficiency to WT levels, while c-di-AMP overexpression (WT::petE-*dacA* strain) did not affect the transformation efficiency (Fig. 1a; Supplementary Fig. S1). These results indicate that the absence of c-di-AMP affects cyanobacterial natural competence negatively, whereas high c-di-AMP does not. A similar result was obtained using the c-di-AMP-null Δ*cdaA* mutant^8^ of *Synechococcus elongatus* (Supplementary Fig. S1), indicating that the c-di-AMP-dependent control of natural competence is a common trait among cyanobacteria.

**Fig. 1:**
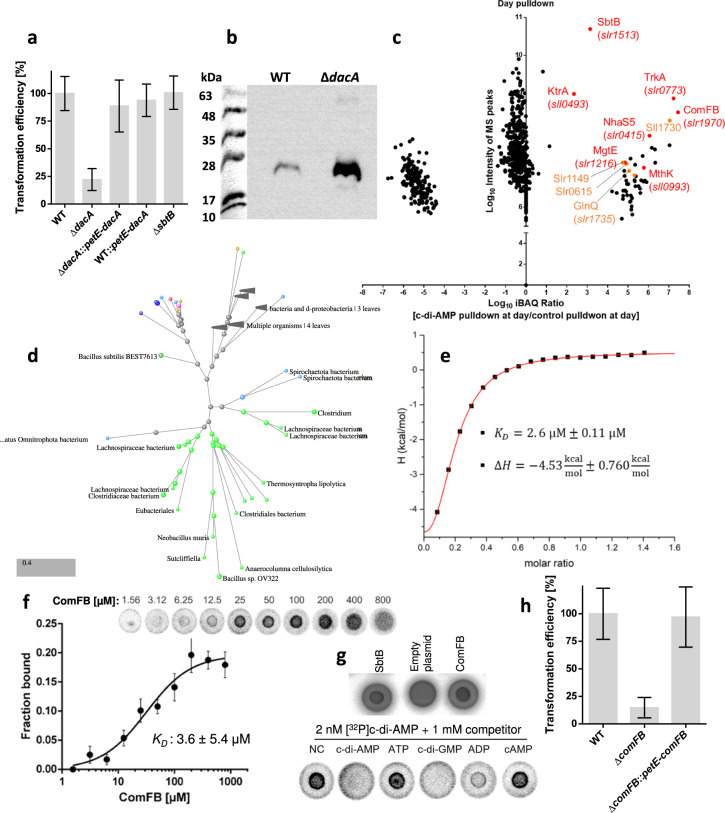
Involvement of c-di-AMP in cyanobacterial natural competence via ComFB signaling protein. **a** Transformation efficiency of WT, ∆*dacA*, *∆dacA::petE-dacA*, WT*::petE-dacA* and ∆*sbtB* strains (see also Supplementary Fig. S1). **b** Immunodetection of PilA1 in the exoproteome of WT and ∆*dacA*. **c** Pulldown experiment using immobilized c-di-AMP and extracts of *Synechocystis* cells grown under day-night cycles, showing the enriched proteins in the day phase. Potential new c-di-AMP receptors are highlighted in orange. **d** Phylogenetic tree showing that ComFB proteins are widespread among different bacterial phyla (detailed tree Supplementary Fig. S4). **e** Dissociation constant (*K*_*D*_) of c-di-AMP binding to ComFB and enthalpy (Δ*H*) are obtained from sigmoidal fitting curve of all ITC experiments with different monomeric ComFB concentrations. **f** DRaCALA assay showing the binding of [^32^P]c-di-AMP to purified ComFB in a concentration dependent manner as indicated. The upper panel shows a representative of one replicate from four technical replicates. The lower panel shows the calculated mean ± SD of the quantification of the bound fraction of [^32^P]c-di-AMP to ComFB from the four replicates and the best fitting curve with the obtained *K*_*D*_ value. **g** DRaCALA competition binding assay showing the competition of [^32^P]c-di-AMP with different nucleotides to bind ComFB. NC refers to no competitor. SbtB and cell extract of *E. coli* harboring an empty plasmid were used as positive and negative control, respectively. **h** Transformation efficiency of WT, ∆*comFB*, and Δ*comFB::petE*-*comFB* strains (see also Supplementary Fig. S1).

Next, we checked how the lack of c-di-AMP affects pilus biogenesis. A proteome analysis of Δ*dacA* compared to WT under day-night cycle, a condition trigger pili biogenesis and natural competence^7^, revealed a strong downregulation of several proteins involved in pilus biogenesis and DNA uptake in Δ*dacA*^4^. These changes were marked by a reduced abundance of PilT1 (Slr0161), PilM (Slr1274), PilN (Slr1275), PilO (Slr1276), and Sll0180 proteins (Supplementary Table S1). The cellular levels of the other pilus machinery proteins were not significantly altered in the Δ*dacA* mutant (Supplementary Table S1). The assembly of a functional pilus requires two motor ATPases, PilB1 and PilT1. PilT1 is located at the pilus base and is required for pili retraction and depolymerization. Therefore, *pilT1* mutant is nonmotile, hyperpiliated and loses natural competence^6^. Similarly, the *pilM*, *pilN* and *pilO* mutants are nonmotile and non-transformable. The PilMNO proteins form the alignment complex^6^, connecting the components of the pilus machinery in the inner and outer membranes by forming a ring-structure in the periplasm. Sll0180 is an accessory protein, needed for PilA1 (Sll1694) glycosylation and S-layer secretion, and thereby the correct assembly of the pilus machinery^6^. Non-functional PilA1 causes a non-transformable phenotype^6^. Notably, our transcriptome analysis^3^ showed a partial downregulation of *pilT1* and *sll0180*, while *pilT2* (*sll1533*) was strongly downregulated in Δ*dacA* (Supplementary Table S2). These findings explain why Δ*dacA* mutant lost the natural competence.

The striking decrease of PilT1 levels in Δ*dacA* suggested a strong defect in pilus assembly and retraction. To test this assumption, we examined negatively stained Δ*dacA* and WT cells by transmission electron microscopy (TEM). While we could detect both thick and thin pili in the WT, only thick pili were obvious in Δ*dacA* (Supplementary Fig. S2). Additionally, Δ*dacA* mutant showed a hyperpiliation phenotype in analogy to *pilT1* mutant. The quantification of the major pilin PilA1 in Δ*dacA* exoproteome revealed an accumulation of PilA1 compared to WT cells (Fig. 1b), further supporting the notion of a c-di-AMP-dependent control of pilus biogenesis and natural competence.

The lack of non-retractable pili explains the inability of the Δ*dacA* to take up DNA. Interestingly, the downregulation of the above-mentioned proteins was not detected in Δ*sbtB* mutant^4^, which lacks the only known cyanobacterial c-di-AMP receptor^2^ (Supplementary Table S3). Furthermore, Δ*sbtB*^9,10^ behaved like the WT in our competence assays (Fig. 1a; Supplementary Fig. S1), suggesting the involvement of an additional, yet unknown, c-di-AMP receptor, required for natural competence. To identify this new c-di-AMP receptor, we performed c-di-AMP-dependent pulldowns using *Synechocystis* cells growing under day-night cycles (Fig. 1c; Supplementary Fig. S3a–c). The identification of several known c-di-AMP targets: SbtB^2,9–11^, as well as the transporters TrkA, KrtA, MthK, MgtE and NhaS5, validated our pulldowns^2^. The Slr1970 protein was also enriched and correlated with the intracellular c-di-AMP levels, where it was more abundant in the day than in the night (Supplementary Fig. S3c). This protein was annotated as ComFB^12^, and we found that ComFB proteins are widespread among different bacterial phyla (Fig. 1d; Supplementary Fig. S4), implying a fundamental role in cell physiology. In *Bacillus, comFB* forms an operon with *comFA* and *comFC*, which are known to be involved in DNA uptake^12^. In cyanobacteria, *comFB* forms an operon with *hfq*^6^, which is also involved in DNA uptake and motility (Supplementary Fig. S5), strongly suggesting a potential function of ComFB in natural competence.

To validate ComFB as a novel c-di-AMP-binding protein, we used several biophysical methods. Size exclusion chromatography coupled to multiangle light scattering showed a species of 40.5 kDa (Supplementary Fig. S6a), indicating that ComFB protein is dimeric (theoretical monomer mass 20.4 kDa). Using isothermal titration calorimetry (ITC)^2,10^, we found that c-di-AMP binds endothermically to ComFB with high affinity of a *K*_*D*_ 2.6 ± 0.11 μM (Fig. 1e; Supplementary Fig. S6b), while no binding was observed for ATP, ADP, AMP, cAMP, and cGMP (Supplementary Fig. S7), indicating that ComFB binds c-di-AMP specifically. This result was confirmed using nanoDSF and thermal shift assays (Supplementary Figs. S8, S9), where c-di-AMP thermally stabilized ComFB in a concentration-dependent manner. Moreover, DRaCALA titration assays revealed strong binding of [^32^P]c-di-AMP to ComFB with a *K*_*D*_ of 3.6 ± 5.4 µM (Fig. 1f). In the competition assays, the unlabeled c-di-AMP competed with [^32^P]c-di-AMP for binding to ComFB, which was not the case for ATP, ADP, and cAMP, confirming that c-di-AMP binding to ComFB is specific. However, this assay revealed that ComFB could additionally bind c-di-GMP, as c-di-GMP efficiently competed with [^32^P]c-di-AMP (Fig. 1g). Remarkably, a recent study showed that the ComFB homolog (named CdgR) controls cell size by binding c-di-GMP^13^ in the multicellular cyanobacterium *Nostoc*, which is regarded as being not naturally competent, implying that ComFB or CdgR might play different roles in multicellularity lifestyle.

To ascertain whether c-di-AMP binding to CdgR is also of physiological relevance, we performed a c-di-AMP-dependent pulldown but using *Nostoc* cell extract (Supplementary Fig. S3d). Indeed, we identified the ComFB homolog (CdgR; Alr3277) as one of the highly enriched proteins along with other known c-di-AMP receptor proteins^2^. This result further confirms that both ComFB and CdgR specifically bind both cyclic di-nucleotides in both organisms. Additionally, ComFB was found to bind c-di-GMP with comparable affinity (1.7 ± 0.5 µM) to that of c-di-AMP^13^. The existence of a crosstalk between c-di-AMP and c-di-GMP on ComFB awaits, however, further investigation. Crosstalk between second messenger nucleotides is perhaps a more common phenomenon than so far realized^2,9^ (see Supplementary Discussion).

To rule out that Δ*dacA* transformation deficiency (Fig. 1a) is due to a downstream effect on the intracellular c-di-GMP content, which is known to regulate motility-related functions^1^, we measured the c-di-GMP levels. The c-di-GMP levels were comparable between Δ*dacA* and WT cells within the light/dark phases (Supplementary Fig. S10), thus confirming that DNA uptake is influenced by c-di-AMP specifically.

To clarify whether ComFB plays a role in natural competence, we created a Δ*slr1970* deletion mutant (Δ*comFB*; Supplementary Fig. S11). Like Δ*dacA*, Δ*comFB* showed reduced transformation efficiency as compared to the WT and Δ*sbtB*^10,11^ cells (compare Fig. 1h with a; Supplementary Fig. S1c). Complementation of Δ*comFB* restored the competence phenotype (Fig. 1h). Interestingly, no impairment in DNA uptake was observed for Δ*sbtB*^10,11^, which lacks another c-di-AMP receptor protein and showed a similar transformation efficiency to the WT cells (Fig. 1a; Supplementary Fig. S1c). These results further support that the natural competence depends on c-di-AMP-signaling and is controlled by a pathway that involves ComFB as a c-di-AMP-receptor. In contrast to Δ*sbtB*^2,9,11^, Δ*comFB* did not show any impairment under diurnal growth (Supplementary Fig. S12), supporting the notion that c-di-AMP plays different signaling functions through binding to different receptors.

To gain insights into the molecular basis of how ComFB controls the natural competence, we carried out a comparative proteome analysis of Δ*comFB* mutant (Supplementary Fig. S13). Surprisingly, PilA1 (Sll1694) was the most upregulated protein in Δ*comFB*, implying a hyperpiliation phenotype like Δ*dacA* (Fig. 1b; Supplementary Fig. S2). Also, Sll1693 (methyl transferase potentially involved in PilA1 and PilA2 methylation) and Sll1696, which are part of *pilA1* operon (*sll1693–sll1696*), showed upregulation. The minor pilin PilX2 (Slr0442) was also upregulated, further supporting the hyperpiliation hypothesis. The S-layer protein (Slr1704) and the outer membrane porin (Sll1550), which are required for the cell envelop and the correct assembly of pilus machinery, were also upregulated in Δ*comFB* mutant. Moreover, the Deg protease (Sll1679), Sll0141 and Sll1581 were also upregulated in Δ*comFB* mutant. The Deg protease (Sll1679) is known to be involved in motility and piliation^6^, while the Sll0141 is an accessory protein needed for pilin glycosylation and secretory machinery^6^ and the Sll1581 is important for the production of cell-surface exopolysaccharides.

Additionally, PixJ1 (Sll0041)^6^ and PixL (Sll0043)^6^ of TaxD1, and PilJ (Sll1294) and CheA (Sll1296)^6^ of TaxD2, which are motility-related proteins and involved in phototaxis^6^, were down-regulated in Δ*comFB*. Consistent with PilA1 upregulation, it was reported previously that Δ*pixL* mutant shows *pilA1* upregulation. The Δ*pilJ* mutant was shown previously to be non-transformable and non-motile. Moreover, several proteins (e.g., Sll0445, Sll0446, Slr0362, and Slr0442) which are under the control of LexA or SyCRP1/2, transcription factors known to regulate motility and pilus biogenesis^6^, were deregulated in Δ*comFB* mutant. In analogy to Δ*dacA*, we detect a downregulation in PilN, which is part of *pilMNO* operon, explaining the reduced transformability of both Δ*dacA* and Δ*comFB* mutants. To further confirm this result, we analyzed the transcript levels of *pilM* using RT-PCR. In fact, *pilM* was downregulated in both Δ*comFB* and Δ*dacA* mutants (Supplementary Fig. S14a). Moreover, we could not detect *comFB* mRNA in Δ*comFB*, while *hfq* showed a normal expression in all strains. Since the levels of *hfq* mRNA were similar to that of the WT, we can safely assume that *comFB* mutation does not cause a polar effect on the upstream *hfq* gene, which is required for pilus assembly^6^, highlighting the specific effect of *comFB* and *dacA* mutations on *pilMNO* operon. As a negative control, we checked for *pilB1* mRNA, which did not change.

To test whether Δ*comFB* causes hyperpiliation analogs to Δ*dacA*, we examined negatively stained Δ*comFB* cells by TEM. Indeed, Δ*comFB* cells were hyperpiliated (Supplementary Fig. S2), and PilA1 quantification revealed a strong accumulation of PilA1 in Δ*comFB* similar to Δ*dacA* (Supplementary Fig. S14b, c). Altogether, these results confirm that c-di-AMP and ComFB are involved in a similar pathway with both being required for pilus biogenesis and natural competence.

Finally, to determine whether the cellular role of ComFB is conserved among cyanobacteria, we created a Δ*comFB* mutant in *S. elongatus* (*Synpcc7942_1924*; Supplementary Fig. S11). The DNA uptake was impaired in this strain (Supplementary Fig. S1e, f), confirming a conserved role for ComFB in natural competence. To gain insight into the pathways that ComFB could coordinate in *S. elongatus*, we checked for co-fitness scores of an Random Barcode Transposon Insertion Site Sequencing (RB-TnSeq) library, which indicates likelihood that two genes participate in similar pathways and respond alike under different growth conditions^14^ (see Supplementary Discussion). Genes, which possess co-fitness values > 0.75 in the RB-TnSeq library, are considered to possess robust co-fitness and likely to participate in similar pathways. *S. elongatus* Δ*comFB* mutant showed a very high co-fitness (0.85–0.98) for mainly genes involved in natural competence and pilus machinery, including *pilA, pilA2, pilB1, pilC*, and *rntAB* (*Synpcc7942_2484–2486*) operon (Supplementary Fig. S15). Similar to *Synechocystis* Δ*comFB*, *S. elongatus* Δ*comFB* showed also a strong association with *pilMNOQ* operon (*Synpcc7942_2450–2453*). Also, several regulatory genes of pilus biogenesis (e.g. *hfq*, *sigF1* and *esbA*) showed strong co-fitness association with *S. elongatus* Δ*comFB* mutant^15^. This result further confirms that ComFB is a new player in controlling cyanobacterial natural competence and pilus biogenesis.

In conclusion, our results show that the regulation of pili biogenesis and natural competence is a new unexplored role of c-di-AMP, which requires the receptor protein ComFB. In a broader context, natural competence is a primary mode of horizontal gene transfer, which plays an important role in spreading multidrug resistance. It would therefore be highly interesting to determine whether the influence of c-di-AMP and ComFB signaling on DNA uptake and/or motility-related functions extends to other bacteria, especially those of clinical relevance. Collectively, we identified ComFB as a novel widespread c-di-NMP-receptor, which turned out to be a pivotal competence-accessory protein, at least in cyanobacteria, regulating the pili biogenesis.

## Supplementary information

Supplementary Information

Supplementary Tables
