# Supplementary material for: The second messenger c-di-AMP controls natural competence via ComFB signaling protein
Source: Cell Discov. 2025 Aug 1;11:65. doi: 10.1038/s41421-025-00816-x (PMC12313861; doi:10.1038/s41421-025-00816-x)
Supplement: Supplementary file 1 — Supplementary Information [file 41421_2025_816_MOESM1_ESM.pdf]

Supplementary Information for

Title: The second messenger c-di-AMP controls natural competence via ComFB signaling protein

Sherihan Samir[1,4,§], Sofía Doello[1,§], Andreas M. Enkerlin[2], Erik Zimmer[1], Michael Haffner[1], Teresa Müller[1], Lisa Dengler[1], Stilianos P. Lambidis[1], Shamphavi Sivabalasarma[3], Sonja-Verena Albers[3], Khaled A. Selim[1,2,*]

[1] Interfaculty Institute of Microbiology and Infection Medicine, Tübingen University, Tübingen, Germany

[2] Microbial Biochemistry Group, Institute of Phototrophic Microbiology, Heinrich-Heine University Düsseldorf, 40225 Düsseldorf, Germany

[3] Molecular Biology of Archaea, Freiburg University, Freiburg, Germany

[4] Microbiology Department, Faculty of Science, Ain Shams University, Cairo, Egypt

[§] These authors contributed equally to this work

***For correspondence:** Khaled A. Selim (khaled.selim@uni-duesseldorf.de)

This PDF file includes:

- Supplementary Text and Discussion
- Materials and Methods
- Supplementary Fig. S1 to S18
- Legend for Supplementary Tables S1 to S5
- Supplementary References

Other supporting materials for this manuscript include the following:

Supplementary Tables S1 to S5 (separate Excel files)

Supplementary Text and Discussion

Second messengers are small molecules involved in regulating many processes in all kinds of organisms (Yoon & Waters 2021). Cyclic di-AMP is one of the recently discovered di-nucleotide-type second messengers only present in prokaryotes (Stülke & Krüger 2020; He et al. 2020; Yin et al. 2020; Mantovani et al. 2023). Its functions have been mainly studied in firmicutes, where it plays an important role in osmo-adaptation by controlling potassium homeostasis and influencing transcription of genes related to osmoregulation and cell wall metabolism (Stülke & Krüger 2020; He et al. 2020; Yin et al. 2020; Herzberg et al. 2023; Nelson et al. 2013; Ren & Patel 2014; Foster et al. 2024). In cyanobacteria, c-di-AMP controls additional processes, including the diurnal metabolism via its binding to the carbon control protein SbtB to regulate glycogen metabolism (Rubin et al. 2018; Selim et al. 2021a). Also, it seems to be implicated in controlling carbon/nitrogen homeostasis, osmoregulation, ion homeostasis, photosynthesis, redox balance, and proteome stability through yet unknown mechanisms (Mantovani et al. 2022; Haffner et al. 2023b). Recently, a role for c-di-AMP in controlling natural competence has been speculated in *Streptococcus pneumoniae*, although also through unknown mechanism (Zarrella et al. 2020). Here, we revealed that c-di-AMP controls the cyanobacterial natural competence through ComFB receptor protein, widespread in bacterial phyla.

Natural competence is a conserved mechanism of horizontal gene transfer that permits massive genetic variation and genomic plasticity via uptake of extracellular DNA, and it is the main cause of spreading antibiotic resistance and acquisition of virulence factors (Gibson & Venning 2023; Ellison et al. 2018). This process involves a contractile pilus system and an assemblage of competence-accessory proteins (Taton et al. 2020; Ellison et al. 2018; Chen et al. 2020). In cyanobacteria, natural competence is under circadian clock control, with pili biogenesis occurring during the day phase and competence being induced with the onset of the night, coinciding with the peak of the circadian cycle (Taton et al. 2020). In the cyanobacterium *Synechocystis* sp. PCC 6803, the cellular levels of c-di-AMP and the transcription of its synthesizing di-adenylate cyclase gene (*dacA*), also follow a circadian rhythm: They decline during the night and increase sharply at the onset of the day (Selim et al. 2021a).

Our results indicate that the absence of c-di-AMP affects cyanobacterial natural competence negatively, whereas high c-di-AMP does not. The proteome analysis of Δ*dacA* mutant (Haffner et al. 2023b) revealed a strong downregulation of PilT2, PilMNO and Sll0180 proteins, which are involved in pilus biogenesis and DNA uptake (Cengic et al. 2018; Bhaya et al. 2000; Okamoto & Ohmori 2002; Chang et al. 2016; Conradi et al. 2020; Chen et al. 2020; Yoshihara et al. 2001; Gonçalves et al. 2018). We were also able to confirm the downregulation of *pilM*

on transcriptional levels (Supplementary Fig. S14a). The *pilT1* mutant is nonmotile, hyperpiliated and loses natural competence. In analogy to Δ*pilT1*, Δ*dacA* mutant showed a hyperpiliation phenotype (Supplementary Fig. S2), consistent with the striking decrease of PilT1 levels in Δ*dacA* (Cengic et al. 2018; Bhaya et al. 2000; Okamoto & Ohmori 2002). These findings explain why Δ*dacA* cells lost their natural competence.

In a search for a c-di-AMP receptor protein, which could propagate and transduce the c-di-AMP signal, we identified a protein possessing a competence factor B domain (domain PF10719 in the Pfam database) (Mistry et al. 2021) and annotated as ComFB in the NCBI's RefSeq database (Haft et al. 2023). The enrichment of ComFB correlated with the intracellular levels of c-di-AMP, where ComFB was more abundant in the day than in the night pulldown (Supplementary Fig. S3c). This indicates that ComFB abundance follows the same pattern as the c-di-AMP synthesis with an increase during the day and a decrease at night (Selim et al. 2021a). We found that ComFB proteins are widespread among different bacterial phyla (Fig. 1d and Supplementary Fig. S4; Samir et al. 2024), implying a fundamental role in cell physiology. In cyanobacteria, *comFB* forms an operon with *hfq,* which is also involved in DNA uptake and motility (Supplementary Fig. S5) (Dienst et al. 2008; Schuergers et al. 2014; Oeser et al. 2021; Conradi et al. 2020). Like Δ*dacA,* Δ*comFB* showed hyperpiliation phenotype (Supplementary Fig. S2 and Supplementary Fig. S14b,c) and reduced transformation efficiency (Fig. 1h and Supplementary Fig. S1) as compared to the WT and Δ*sbtB* cells of another c-di-AMP receptor protein (Selim et al. 2021a).

Surprisingly, we found that ComFB could potentially bind both c-di-AMP and c-di-GMP. In fact, a recent study showed that the ComFB homolog in multicellular cyanobacteria, which is regarded as being not naturally competent (Schirmacher et al. 2020), controls cell size by binding c-di-GMP (Zeng et al. 2023). The existence of a crosstalk between c-di-AMP and c-di-GMP on ComFB awaits, however, further investigation. Crosstalk between second messenger nucleotides is perhaps a more common phenomenon than so far realized. Recently, it was found that the second messengers c-di-GMP and (p)ppGpp reciprocally control *Caulobacter crescentus* growth by competitive binding to a metabolic switch protein, SmbA (Shyp et al. 2021). SbtB plays a similar role in cyanobacterial physiology and binds both cAMP and c-di-AMP (Selim et al. 2018, 2021a, 2023; Selim & Alva 2024; Forchhammer et al. 2022). Likewise, the mycobacterial transcription factor DarR, which is regulated by c-di-AMP-binding, was found to be regulated as well through cAMP-binding (Schumacher et al. 2023). Additionally, crosstalk between cyclic guanosine and adenosine second messengers is also known, as the CRP-Fnr transcription factors are known to bind both cAMP and cGMP, being only active in the cAMP-bound form in *E. coli*, while both cyclic nucleotides mediate the CRP activation in *Sinorhizobium meliloti* (Krol et al. 2023; Werel et al. 2023). Furthermore, both of cAMP and

cGMP were found also to bind and modulate the activity of the AphA phosphatase in *E. coli* and *Haemophilus influenzae* (Kronborg & Zhang 2023).

In accordance with the hyperpiliation phenotype (Supplementary Fig. S2), Δ*comFB* showed upregulation of proteins of PilA1 operon (*sll1693*-*sll1696*), including PilA1 (Sll1694; Supplementary Fig. S13) (Sergeyenko & Los 2000; Singh et al. 2005; Linhartová et al. 2021). Similarly, the minor pilin PilX2 (Slr0442; Oeser et al. 2021) was also upregulated. In analogy to Δ*dacA* mutant, *pilMNO* operon showed a downregulation on transcriptional (Supplementary Fig. S14a) and protein levels (Supplementary Fig. S13) explaining the reduced transformability of both Δ*dacA* and Δ*comFB* mutants (Fig. 1a,h and Supplementary Fig. S1).

The cellular function of ComFB seems conserved among unicellular cyanobacteria as Δ*comFB* deletion mutants showed similar phenotype in *S. elongatus* and *Synechocystis* (Supplementary Fig. S1). The RB-TnSeq tuned out to be a powerful high-throughput genetic screening technique to identify the function of genes by assessing the impact of gene disruptions on cellular fitness under different conditions (Wetmore et al. 2015; Price et al. 2018). The RB-TnSeq library of *S. elongatus* was grown under different stress conditions and the abundance of the mutants in the library is monitored by the barcodes sequencing, allowing to determine the fitness of each mutant under all stress conditions. *S. elongatus* Δ*comFB* mutant showed a very high co-fitness for mainly genes involved in natural competence and pilus machinery (Suban et al. 2022, 2024; Taton et al. 2020), including *pilA, pilA2, pilB1, pilMNOQ*, *pilC, hfq, sigF1* and *rntAB* (Supplementary Fig. S15), supporting a crucial role for ComFB as a new player in controlling cyanobacterial natural competence and pilus biogenesis. Currently, the open questions are how mechanistically ComFB exerts its function to regulate pilus biogenesis and what is the influence of c-di-NMP inputs on ComFB output, which wait for further investigation.

Materials and methods (Full protocols):

Protein production and purification: All the plasmids and primers used in this study are listed in (Supplementary Table S5). The *slr1970* (encoding ComFB) and *slr1513* (encoding SbtB) from *Synechocystis* sp. PCC 6803 were cloned into the pET-28a vector with Gibson assembly, thereby incorporating a C-terminal His_8-tag. Positive clones were selected on 50 μg/mL kanamycin agar plates. The expression and purifications of His_8-ComFB, His_6-DisA and His_8-SbtB proteins were achieved as described previously (Selim et al. 2019, 2021b). The proteins were recombinantly produced in *E. coli* strain LEMO21 (DE3) by overnight induction at 20 ℃ using 0.5 mM IPTG. Cells were lysed by sonication and the soluble proteins (ComFB, DisA or SbtB) were purified by immobilized metal affinity chromatography (IMAC) using Ni^{2+}-Sepharose resin (cytiva™), followed by size exclusion chromatography (SEC) using a Superdex 200 Increase 10/300 GL column (GE HealthCare). Protein purity was assessed by Coomassie-stained SDS-PAGE, and protein concentrations were determined using Bradford assay. Analytical SEC coupled to multi-angle light scattering (SEC-MALS) was conducted as described previously to calculate the molar mass of ComFB protein, whereas SbtB was used as a control (Selim et al. 2019, 2020; Walter et al. 2019).

Synthesis of [^{32}P]c-di-AMP: The pGP2563 plasmid (pET19b-based; kindly provided by Jörg Stülke), expressing an active di-adenylate cyclase His_6-DisA from *Bacillus subtilis* (Mehne et al. 2013), was used to express and purify DisA. Twenty μM or 50 μM DisA were incubated in DisA reaction buffer (40 mM Tris/HCl [pH 7.5], 100 mM NaCl and 10 mM $MgCl_2$) with 1 mM ATP at 30 ℃ overnight with 300 rpm shaking. Samples were centrifuged for 10 min at 14.000 rpm to remove precipitated protein. Then, the supernatant was filtered through Amicon® Ultra – 0.5 mL 10 kDa cutoff centrifugal filters (Merck KgaA; Darmstadt, Germany). To test the enzymatic efficiency of DisA before synthesizing [^{32}P]c-di-AMP, DisA was incubated with unlabeled ATP and 15 µl of the reaction product was analysed by thin layer chromatography (TLC; POLYGRAM CEL300 PEI plates) (Macherey-Nagel GmbH & Co. KG, Düren, Germany) using a running buffer of [1 vol. Saturated NH_4SO_4 (4.2 M) and 1.5 volumes 1.5 M KH_2PO_4 (pH 3.6)] (Supplementary Fig. S16). Via injecting 5 µl of the reaction product, the activity of DisA was further confirmed by LC-MS (ESI-TOF mass spectrometer; MicrO-TOF II, Bruker) connected to Ultimate 3000 HPLC system (Dionex) on C18 column (Phenomenex, 150×4.6 mm, 110 Å, 5 µm) and using a flow rate of 0.2 mL/min and a 45 min program (for 5 min, 100 % buffer A (0.1 % formic acid with 0.05% ammonium formate), then 30 min of a linear gradient to 40 % buffer B (100 % acetonitrile), and 10 min of column re-equilibration with 100 % buffer A). Data are presented as extracted ion chromatograms (EICs) for ATP and c-di-AMP (Supplementary Fig. S16). Finally, 250 µCi radiolabeled [^{32}P]c-di-AMP was synthesized from

[α-^{32}P]ATP (6000 mCi/µmol) by using His_6-tagged DisA by Hartmann Analytic GmbH (Braunschweig, Germany).

***In vitro* protein-ligand binding assays:** Binding of recombinantly produced ComFB to c-di-AMP (or other nucleotides: ATP, ADP, AMP, cAMP and cGMP) was analyzed *in vitro* by isothermal titration calorimetry (ITC), thermal shift assay (TSA), and differential scanning fluorimetry (nanoDSF), as described previously (Lapina et al. 2018; Haffner et al. 2023a; Mantovani et al. 2024). For ITC and TSA, both ComFB and c-di-AMP were dissolved in binding buffer (50 mM Tris/HCl, pH 8.0, 300 mM NaCl, 0.55 mM EDTA). ITC measurements were conducted on a MicroCal PEAQ-ITC instrument (Malvern Panalytical), at 25 ℃, with a reference power of 10 µcal/s. Different ComFB protein concentrations in the range of 60-172 µM were titrated against 0.5 or 1 mM c-di-AMP. A control experiment was recorded by titration of c-di-AMP over a cell filled with buffer, to measure the dilution heat of c-di-AMP. Data were analyzed using one set of binding sites model with the MicroCal PEAQ-ITC Analysis Software (Malvern Panalytical) to calculate the dissociation constant K_D for monomeric ComFB protein of 60, 72, 134 and 172 µM. The dilution heat of the control ITC buffer/c-di-AMP experiments were subtracted from the ComFB/c-di-AMP runs. For reproducibility, different patches of ComFB protein purifications were used in different ITC experiments. TSA measurements were conducted on an iQ5 Real-Time PCR detection system (Bio-Rad). ComFB (10-39 µM) and 0-1.2 mM c-di-AMP were mixed in different ratios, with the addition of 10x SYPRO Orange. All conditions were measured in triplicate in sealed 96-well plates by following the dye's fluorescence emission over a temperature range of 25 to 99 ℃. Data were analyzed with OriginPro software (OriginLab Corporation) and Python. For nanoDSF (Nanotemper), both ComFB and SbtB proteins were diluted in the ITC buffer and used at 1.5 mg/mL concentration with or without 0.5 mM c-di-AMP. The proteins autofluorescence (350/330 nm ratio), as well as light scattering were measured in a temperature range of 30-99 ℃ to determine the melting curve and the rate of protein unfolding.

Differential Radial Capillary Action of Ligand Assay (DRaCALA): The specificity of c-di-AMP binding to ComFB has been verified using DRaCALA (Differential Radial Capillary Action of Ligand Assay) (Roelofs et al. in 2011) using either *E. coli* cell lysate or purified proteins. For cell lysate, ComFB or SbtB has been overexpressed in *E. coli* LEMO21 (DE3), and cell lysates were used for the DRaCALA assays. In the DRaCALA assays, the cell lysates (with total protein concentration of 20 µg) or purified proteins (ComFB or SbtB) were mixed with 2 nM of a radioactively labeled [^{32}P]c-di-AMP (~ 6000 mCi/µmol) for 15 min at room temperature in a binding buffer of (10 mM Tris/HCl [pH 8.0], 100 mM NaCl and 5 mM $MgCl_2$). In the competition binding assays, the [^{32}P]c-di-AMP was incubated first for 2 mins with the protein or the cell

lysates, before adding 1 mM of unlabeled nucleotides (c-di-AMP, c-di-GMP, ATP, ADP, and cAMP) to the reaction mixture for 15 mins. Finally, 10 µL of each mixture were dropped on a nitrocellulose membrane (Amersham™ ProtanTM 0.2 µm NC, Catalogue No10600001, Cytiva Europe GmbH, Freiburg), which binds to the protein while the free ligands diffuse, thereby a radioactive signal appears at the center of the drop application in case of binding of the [^{32}P]c-di-AMP to ComFB. After drying, the nitrocellulose membranes were transferred into an X-ray film cassette and the imaging plates (BAS-IP MS 2025, 20 x 25 cm, FUJIFILM Europe GmbH, Düsseldorf, Germany) were placed directly onto the nitrocellulose membrane, then the cassettes were closed and incubated overnight. The next day, the plates were imaged with a Typhoon™ FLA9500 PhosphorImager (GE Healthcare). For [^{32}P]c-di-AMP signal quantification, the image analysis software Image Studio Lite Ver 5.2.5 was used. The fraction of bound nucleotide was calculated based on (Roelofs et al. 2011) using the following equation:

$$F_B = \frac{I_{inner} - \left[A_{inner} \times \frac{(I_{total} - I_{inner})}{(A_{total} - A_{inner})}\right]}{I_{total}}$$

In the DRaCALA competition assays, if one of the unlabeled nucleotides binds to the ComFB, it will compete on the same binding sites and will reduce or eradicate the radioactive signal of [^{32}P]c-di-AMP binding to ComFB. SbtB, a known c-di-AMP receptor protein (Selim et al. 2021a), and extract of *E. coli* cells expressing an empty plasmid were used as positive and negative controls, respectively.

Phylogenetic analysis: Phylogenetic analysis was done essentially as described elsewhere (Neumann et al. 2022). Homologous protein sequences to the ComFB full-length protein (Slr1970, amino acids 1-173) and to the ComFB-domain only (amino acids 57-147) were searched against the Ref-Seq Select proteins database, using the NCBI blastp suite. ComFB hits were filtered (expectation value $E \leq 10^{-3}$). The sequences were submitted to multiple sequence alignments (MSA) using COBALT (NCBI). Phylogenetic trees were constructed based on the MSAs and visualized using the iTOL online tool (Letunic et al. 2021).

Construction of mutant strains: The unicellular, freshwater cyanobacterium *Synechocystis* sp. PCC 6803, described in (Selim et al. 2021a), was used as the reference wildtype strain in this study. All plasmids and primers used in this study are listed in (Supplementary Table S5). All constructs used in this study were generated using Gibson assembly. All knockout mutants were generated with homolog recombination using the natural competence of *Synechocystis* sp. PCC 6803, as described previously (Selim et al. 2018).

For generation of knockout deletion mutants, the mutants were constructed by deleting the ORFs *slr1513*, *sll0505*, and *slr1970* (designated *sbtB*, *dacA*, and *comFB*, respectively) and replaced with the erythromycin, kanamycin, and spectinomycin resistance cassette,

respectively. The Δ*sbtB* and Δ*dacA* knockout mutants were created, as described previously (Selim et al. 2018, 2021a). For generation of the knockout mutation in the *slr1970* ORF (designated *comFB*; Supplementary Fig. S11), a synthetic DNA fragment encoding the upstream and downstream regions of *slr1970* (0.5 kb) and the spectinomycin resistant cassette (gBlock, IDT, USA) were cloned into digested pUC19 vector using the Gibson cloning strategy. For complementation, the Δ*dacA*::petE-*dacA*, WT::petE-*dacA* and Δ*comfB*::petE-*comFB* strains were generated by introducing the *dacA* gene (*sll0505*) or *comFB* gene (*slr1970*) under the control of Cu^{2+} inducible promoter P*petE* into respective mutant backgrounds using the self-replicating plasmid pVZ322, as described previously (Selim et al. 2018, 2021b). All the plasmids used to generate the mutants were verified by sequencing and then transformed in *Synechocystis* sp. PCC6803, as described (Selim et al. 2018). All mutants were selected on BG_{11} plates supplemented with proper antibiotics and verified by PCR.

The c-di-AMP-free mutant in the ORF (*Synpcc7942_0263*) of *Synechococcus elongatus* PCC 7942 was created as described previously (Rubin et al. 2018) using a *cdaA*-deletion plasmid (AM5403; kindly gifted from Susan Golden) carrying the spectinomycin/streptomycin resistance gene *aadA*. For creation of Δ*comFB* (*Synpcc7942_1924*) mutant in *S. elongatus*, 50 mL of *S. elongatus* culture with an OD_{750} of 0.7 were centrifuged at 4200 rpm for 20 min at room temperature. The cell pellet was resuspended in 500 µl of fresh BG11 media. 1 µg of the plasmid pUC19-Δ7942*comFB* was added to the cell suspension. This plasmid contains a spectinomycin resistance cassette flanked by the homologous sequences of *Synpcc7942_1924* that allow recombination at the *comFB* locus. This construct was ordered as a gBlock from IDT (San Diego, USA) and inserted into the pUC19 backbone using Gibson assembly. Cells were incubated with the plasmid in the dark for 24 h and then 250 µl of the cell suspension were spread on a HATF membrane (HATF08250, Sigma-Aldrich, Germany) placed on a BG11-agar plate. After 16 h, the membrane was transferred to a BG11-agar plate supplemented with 15 µg/mL of spectinomycin. After 3 days, the antibiotic concentration was increased to 25 µg/mL, and after additional 3 days to 50 µg/mL. Colonies were visible after 12 days.

Transformation assay: Natural transformation competence was assessed with different suicide plasmids, all encoding chloramphenicol resistance and integrating into different places in the *Synechocystis* sp. PCC 6803 genome (Oeser et al. 2021). Briefly, the cells of wildtype (WT) *Synechocystis* or respective mutants (Δ*dacA*, Δ*sbtB*, Δ*comFB*, Δ*dacA*::petE-*dacA*, WT::petE-*dacA* and Δ*comFB*::petE-*comFB*) were cultivated in BG_{11} (50 mL) at 28 °C, constant 50 µE m^{-2} s^{-1} and shaking to an OD_{750} of 0.7, then harvested at 4,000 g for 20 min. Cell pellets were resuspended in 600 µl of BG_{11} and all samples were adjusted to the same OD_{750}. Cells suspensions were transferred to a 1.5 mL tube and 1 µg of different plasmids, containing

chloramphenicol resistance cassette, were added to ensure the reproducibility. The 1.5 mL tubes were covered with aluminum foil and incubated at 28 ℃ for 3 h, then gently inverted and incubated for 3 more h. 0.45 µm HATF membranes (HATF08250, Sigma-Aldrich, Germany) were placed on BG_{11} plates and 200 µl of the cell suspensions were spread on them. Plates were incubated at 28 ℃ and 50 µE m^{-2} s^{-1} for 16 h and membranes were then transferred to BG_{11} plates supplemented with 15 µg/mL of chloramphenicol. After 48 h of incubation at 28 ℃ and 50 µE m^{-2} s^{-1}, membranes were transferred to BG_{11} plates supplemented with 30 µg/mL of chloramphenicol and further incubated until singles colonies were visible. At least three-five biological replicates were used for each strain. Some clones were verified by PCR for the insertion of chloramphenicol cassette into the genome. The natural transformation competence for *S. elongatus strains* (WT, Δ*cdaA* and Δ*comFB* mutants) was done as described for *Synechocystis* but using a plasmid carrying kanamycin resistance cassette.

Exoproteome analysis: *Synechocystis* sp. PCC 6803 wildtype, Δ*dacA* and Δ*comFB* cells were grown in 250 mL of BG_{11} at 28 ℃, constant 50 µE m^{-2} s^{-1} and shaking to an OD_{750} of 0.8. Cultures were spun down at 4,000 g for 20 min and the supernatant was filtered through cellulose nitrate membrane filters (7182-004, Cytiva, Marlborough, MA, USA) and concentrated to 1 mL using Amicon Ultra-15 centrifugal filters with a cutoff of 10 kDa (UFC901024, Sigma-Aldrich). Three biological replicates were prepared for each strain. Immunoblot detection of PilA1 in the exoproteome extracts was done as previously described (Oeser et al. 2021) using α-PilA1 antibody (kindly provided by Roman Sobotka; Linhartová et al. 2014).

Transmission electron microscopy (TEM): Cells (WT, Δ*dacA* and Δ*comFB*), growing BG_{11} at 28℃ under continuous illumination 50 µE m^{-2} s^{-1}, were negatively stained with 2% aqueous uranyl acetate (w/v). Imaging was done with Hitachi HT7800 operated at 100 kV, equipped with an EMSIS Xarosa 20-megapixel CMOS camera (Oeser et al. 2021). Acquired images were analyzed with ImageJ.

Mass spectrometry-based proteomics analysis: The full proteomics analysis of the *Synechocystis* sp. PCC 6803 wildtype, Δ*sbtB*, Δ*dacA* and Δ*comFB* cells, growing under day-night cycles, was done as described previously in (Haffner et al. 2023b). The full proteomics data sets of Δ*sbtB* and Δ*dacA* mutants are described in (Haffner et al. 2023b). The full proteomic data sets of Δ*comFB* mutant is described in this manuscript in details (Supplementary Table S4). For the wildtype (WT) strain and Δ*comFB* mutant, the proteome analysis of three independent replicates were performed and displayed high reproducibility based on protein abundance correlations (Supplementary Fig. S17). More than 1730 proteins

could be identified in our proteome dataset (Supplementary Table S4). The overall protein abundances in the Δ*comFB* mutant was slightly divergent from those of WT cells, as indicated by PCA (Supplementary Fig. S18), indicating profound changes in the proteome as a result of Δ*comFB* mutation.

To identify c-di-AMP receptors, cell extracts of *Synechocystis* cells grown under day-night cycles (condition that triggers pili biogenesis and natural competence) were incubated with immobilized c-di-AMP and bound proteins were identified by mass spectrometry. The pulldown experiments to identify the potential c-di-AMP target proteins were done as described previously (Selim et al. 2021a) using *Synechocystis* sp. (under day and night conditions) and *Nostoc*. sp. PCC 7120 cell extracts.

The determination of intracellular c-di-GMP concentration in wildtype and Δ*dacA* cells was done as described in (Selim et al. 2021a) using mass spectrometry calibrated with $^{13}C_{20}{}^{15}N_{10}$-c-di-GMP and $^{13}C_{20}{}^{15}N_{10}$-c-di-AMP (200 ng/ml each).

Data availability: The mass spectrometry proteomics data have been deposited to the ProteomeXchange Consortium via the PRIDE partner repository with the dataset identifier PXD045008.

Supplementary Figures (S1 to S18)

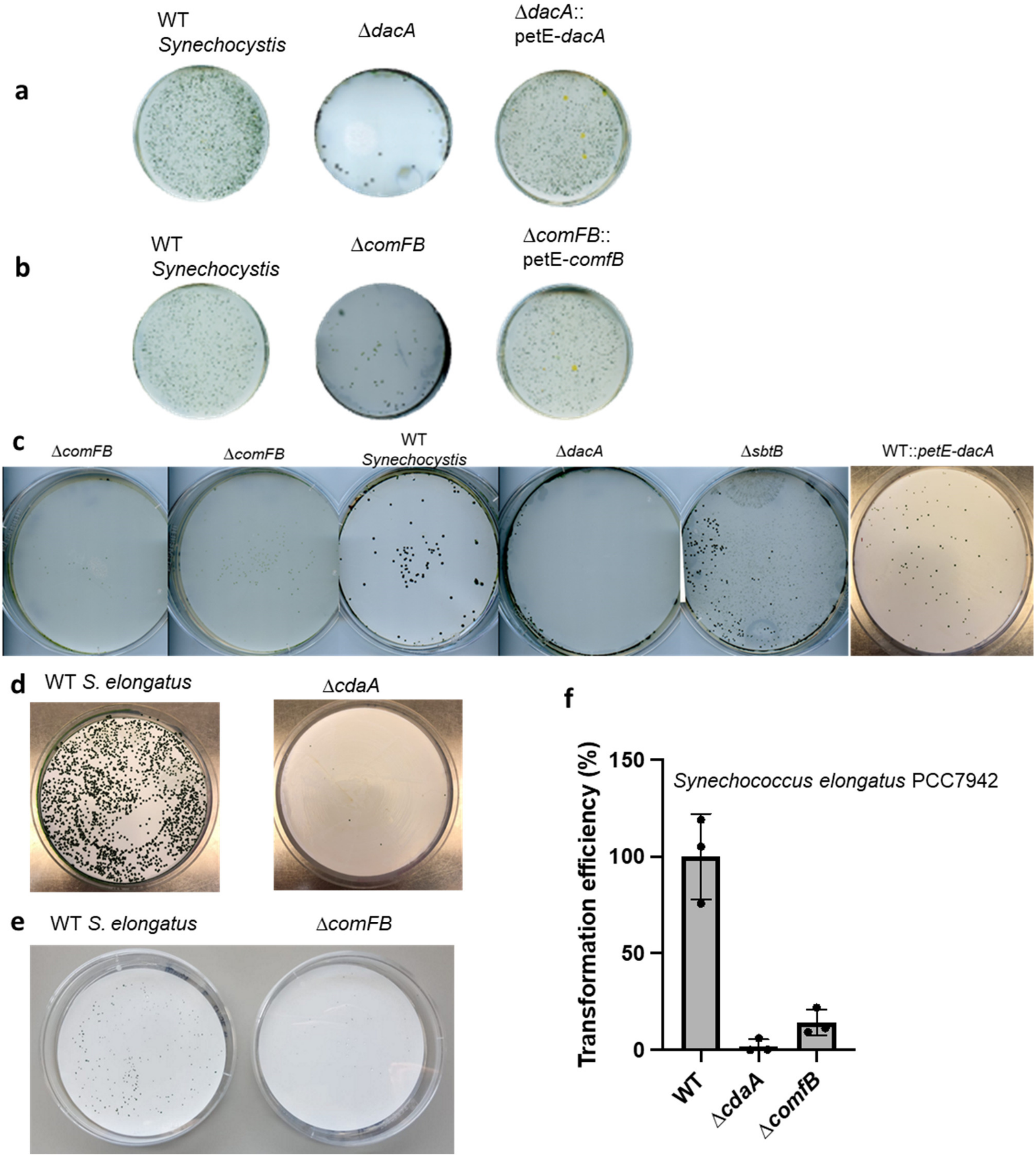

Supplementary Fig. S1. Transformation efficiency of ∆*comFB* and c-di-AMP-free mutants in *Synechocystis* and *Synechococcus elongatus strains.* (a-c) Representative of transformation efficiency in colony forming unites of *Synechocystis* wildtype (WT), ∆*dacA*, *dacA::*petE-*dacA,* ∆*comFB*, and *comFB::*petE-*comFB* strains using different plasmids with chloramphenicol resistance cassette. ∆*sbtB* mutant was used as a negative control, representing another c-di-AMP receptor protein. **(d,e)** Representative of transformation efficiency in colony forming units of *S. elongatus* WT and the mutants of ∆*cdaA* (d) and ∆*comFB* **(e)**. **(f)** Transformation efficiency of *S. elongatus* strains (WT, ∆*cdaA and* ∆*comFB*).

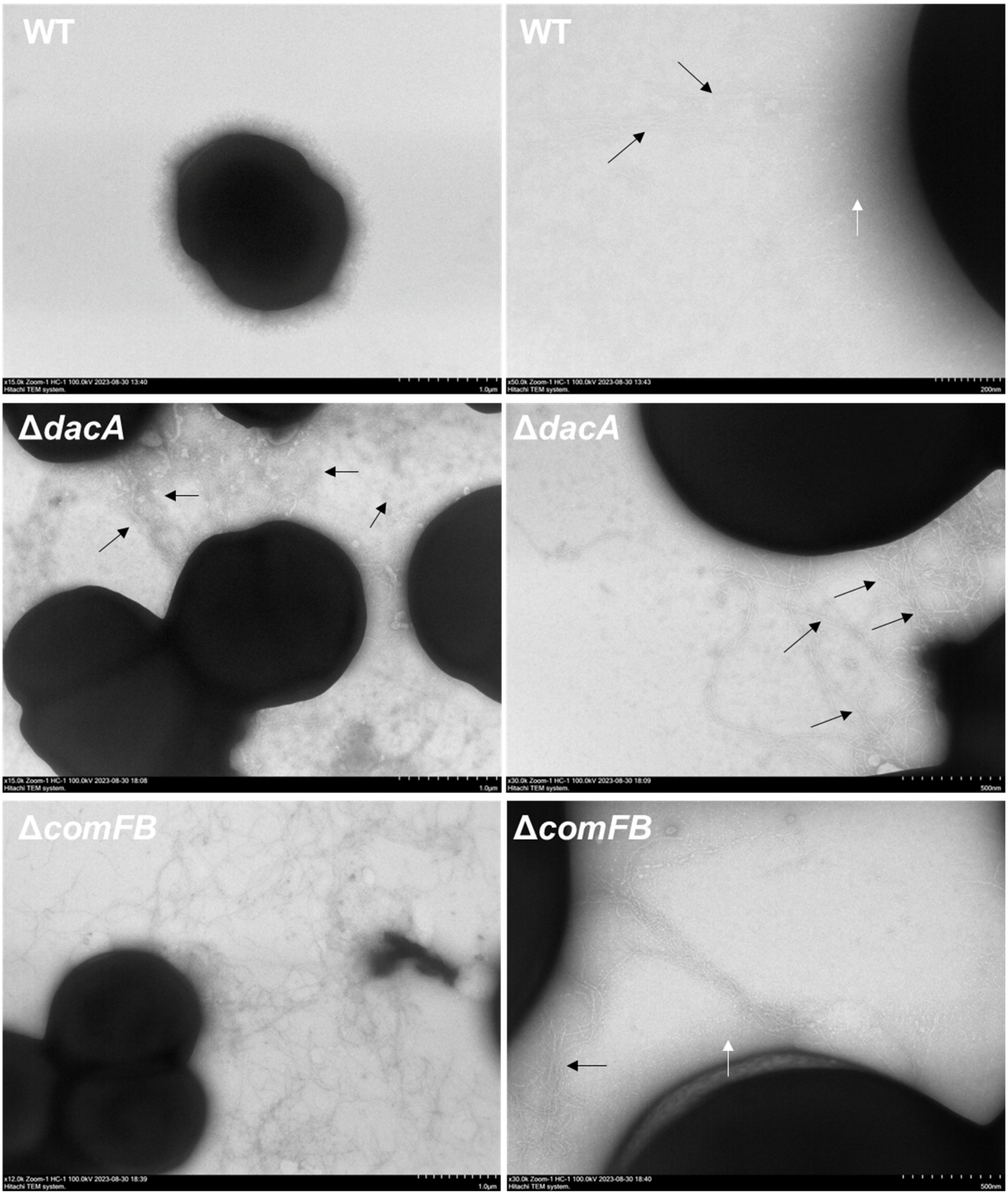

Supplementary Fig. S2. Electron micrographs of negatively stained *Synechocystis* wildtype (WT), Δ*dacA* and Δ*comFB* cells. Whole cells are depicted with 1 □m scale bar and ultrastructural details of pili are shown in 200-500 nm with distinct types of thick pili (black arrow) and thin pili (white arrow). See also additional examples in Supplementary Fig. S2 (*continue*).

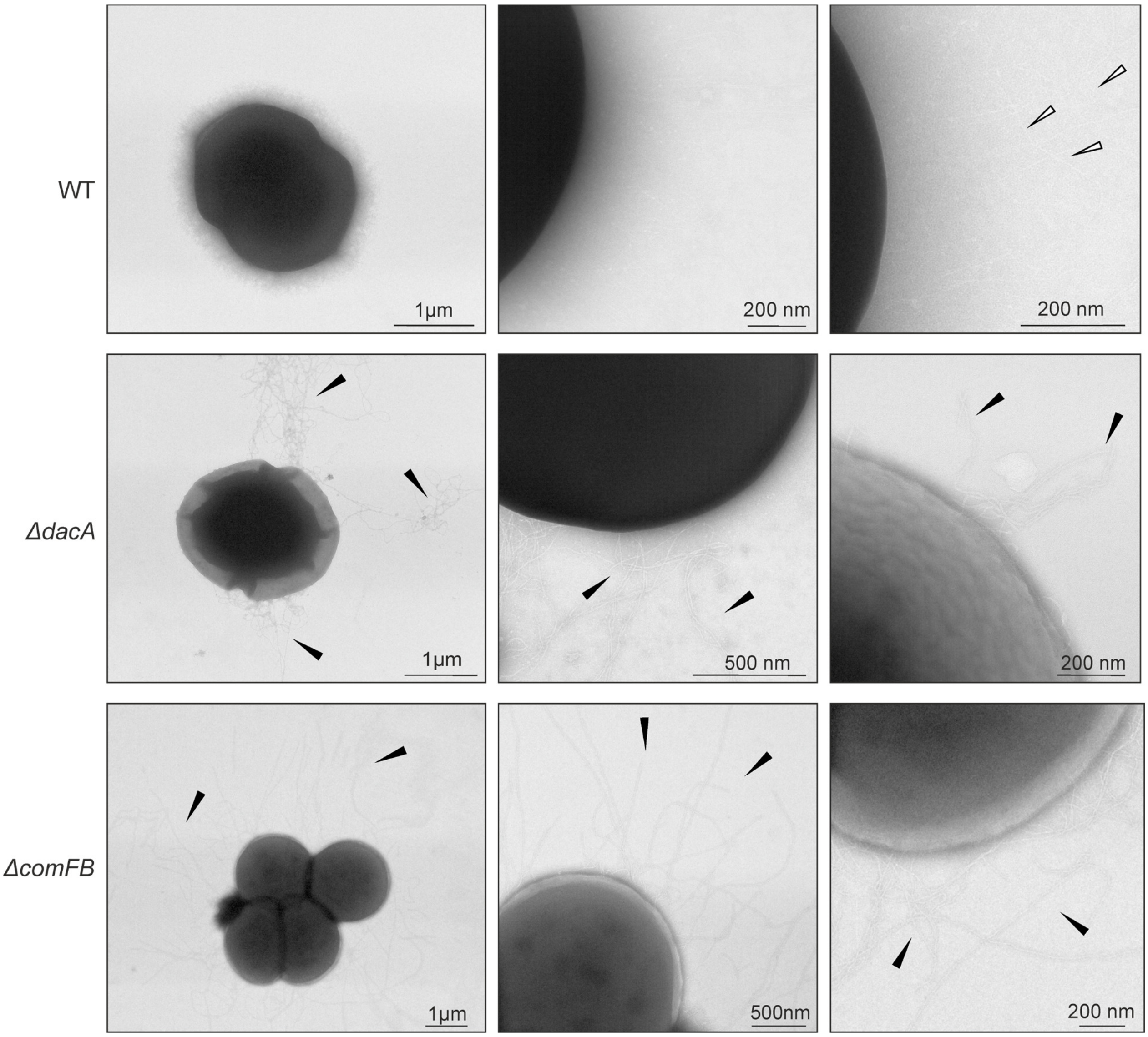

Supplementary Fig. S2 (*continue*): Another example of electron micrographs of negatively stained *Synechocystis* wildtype (WT), Δ*dacA* and Δ*comFB* cells. Whole cells are depicted with 1 µm scale bar and ultrastructural details of pili are shown in 200-500 nm with distinct types of thick pili (black arrow) and thin pili (white arrow).

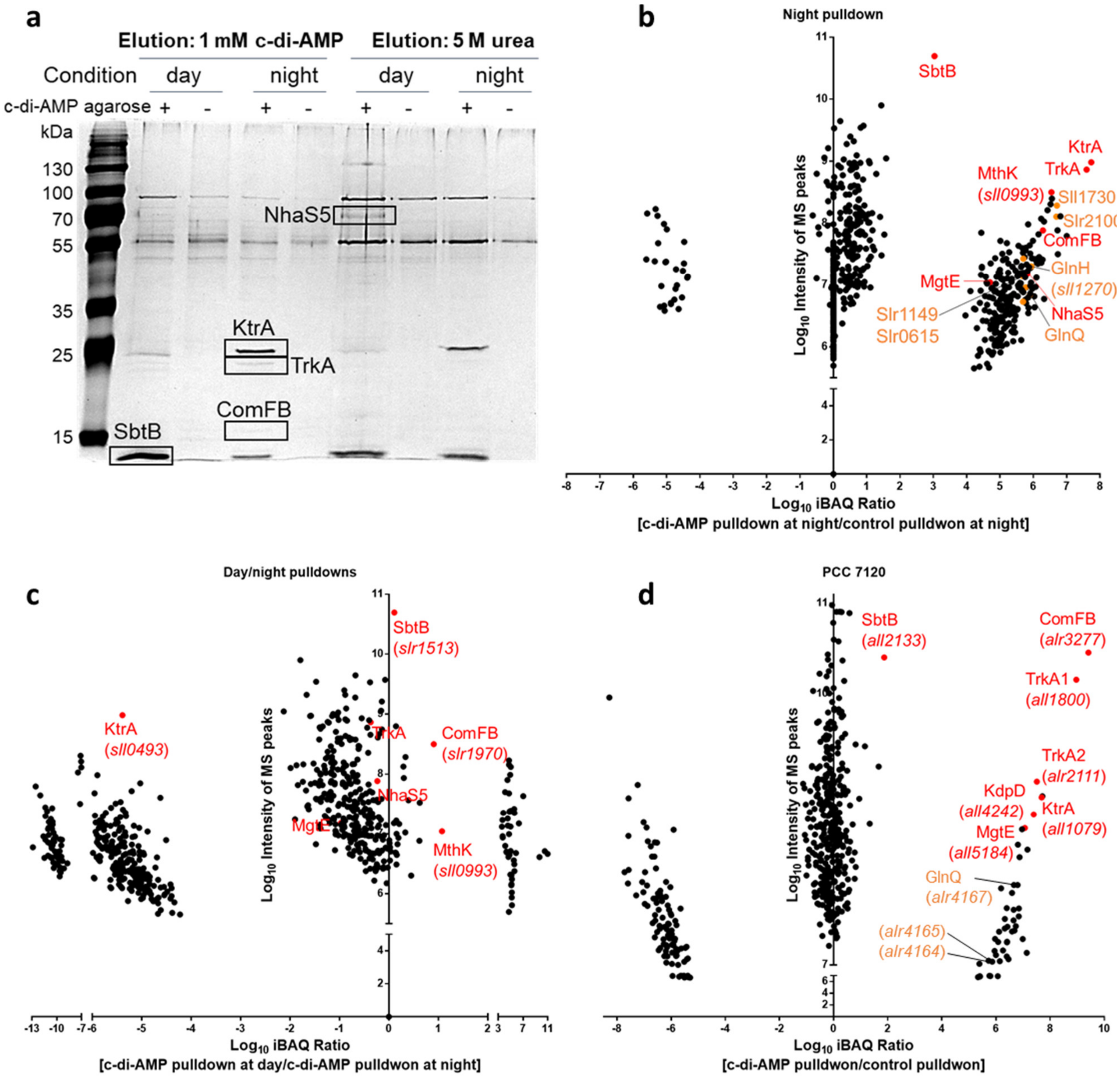

Supplementary Fig. S3. Identification of potential c-di-AMP binding proteins in *Synechocystis* sp. PCC 6803 and *Nostoc* sp. PCC 7120 using immobilized c-di-AMP pulldowns and analyzed by Mass spec. proteomics. (a) SDS-PAGE of c-di-AMP pulldown elution fractions in *Synechocystis* sp. PCC 6803 under day and night conditions, as indicated, with highlight of the potential targets. Elution of bound proteins was achieved by using 1 mM c-di-AMP or 5 mM urea. **(b)** Identification of potential c-di-AMP binding proteins in *Synechocystis* sp. PCC 6803 under night, enriched proteins are highlighted. **(c)** enrichment of ComFB in day pulldown (in Fig. 1c) compared to night pulldown. **(d)** Identification of potential c-di-AMP binding proteins in *Nostoc* sp. PCC 7120, enriched proteins are highlighted in red. **(b-d)** Eluates were analyzed by high accuracy LC-MS/MS to calculate protein enrichment ratios. The identified proteins were sorted by score and refined manually to remove unspecific binning proteins. Significantly enriched proteins were calculated based on Log10 of iBAQ ratio and plotted against the intensity of MS peaks of the identified/defined peptides. The well-known c-di-AMP receptors: SbtB, TrkA, MgtE and KtrC validated our pulldown approach (Selim et al. 2021a). Potential new c-di-AMP receptors are highlighted in orange.

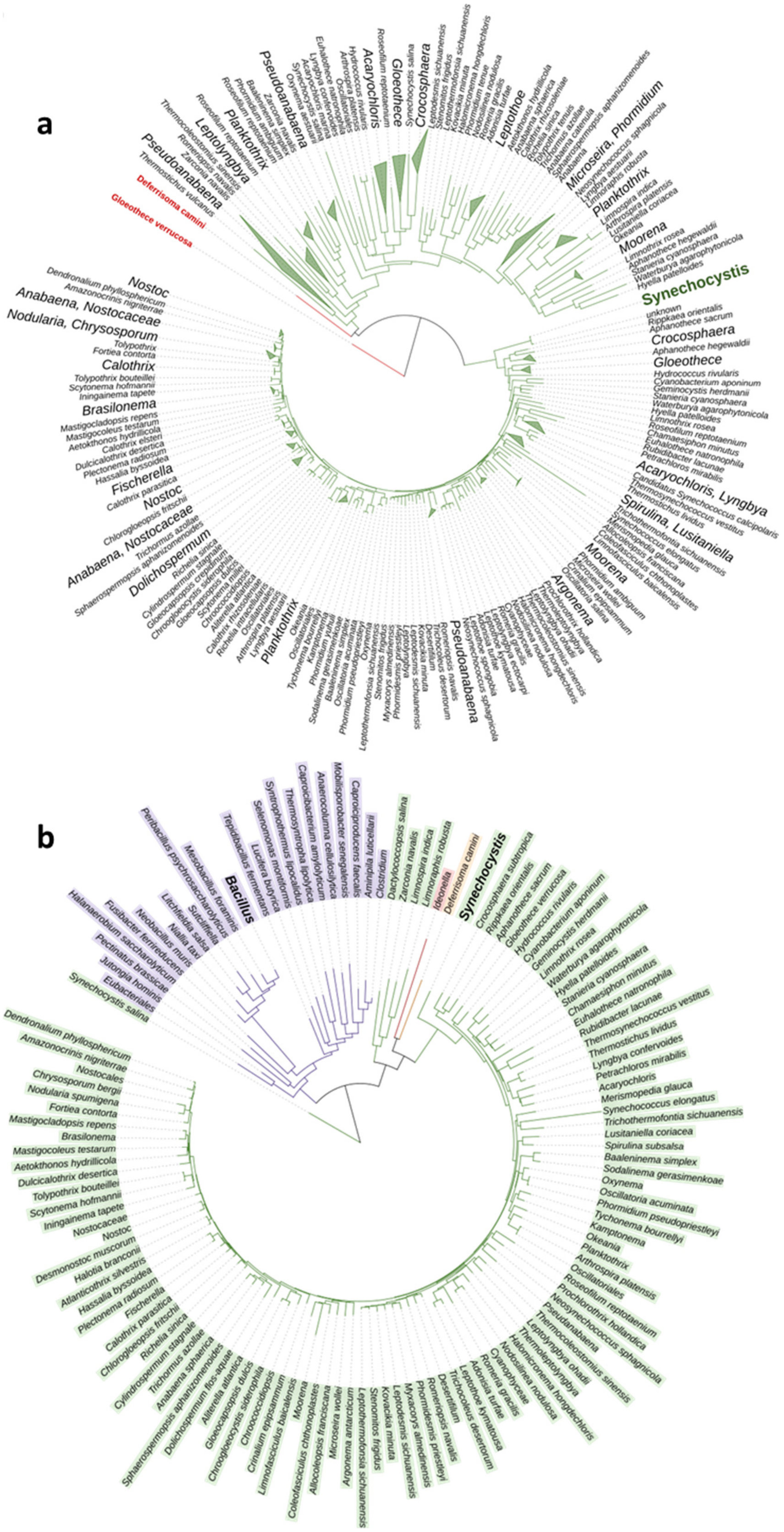

Supplementary Fig. S4. Rooted phylogenetic tree of ComFB homologs. (a) Distribution among cyanobacteria (green). Collapsed clades are shown as triangles. **(b)** Distribution among cyanobacteria (green), firmicutes (violet), ß-proteobacteria (red) as well as other bacteria (orange). Branch lengths represent genetic divergence. Constructed based on a multiple sequence alignment with sequences obtained from a blastp search of the ComFB domain of Slr1970.

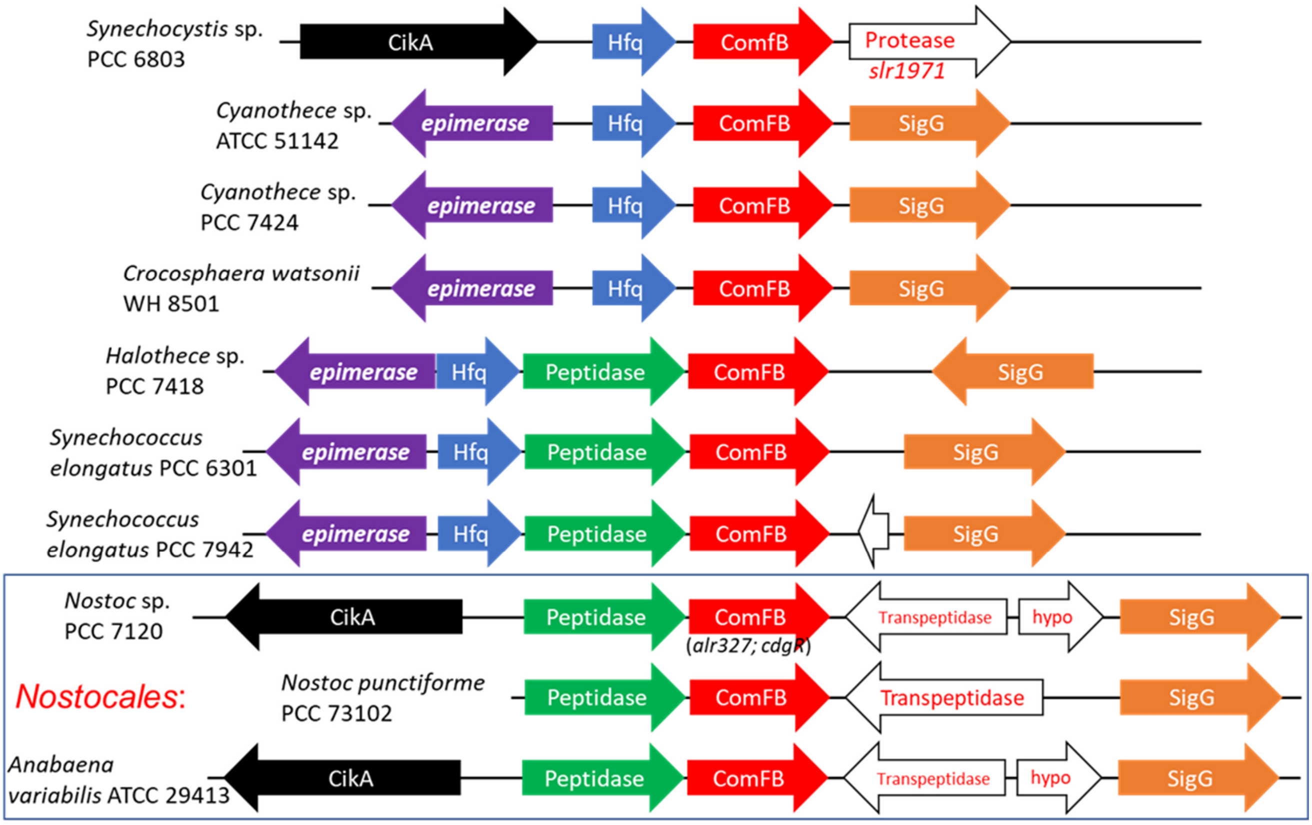

Supplementary Fig. S5. Genomic organisation and conservation of *comFB* homologs (in red) using SEED database in different cyanobacteria species, as indicated. In *Synechocystis* sp. PCC 6803, upstream of *comfB* (*slr1970*; in red) the open reading frames of *hfq* (*ssr3341*; in blue) and *cikA* (*slr1969;* in black) are found. In other cyanobacterial species, the ORFs (open reading frames) encoding for the RNA polymerase sigma factor (SigG; in orange), putative peptidase (in green) and diaminopimelate epimerase (in violet) are found in association with *comfB* as well. Further ORFs which show no strong conservation (e.g. L,D-transpeptidase) or of hypothetical proteins (hypo) are coloured white.

The ORF coding for an orthologue of the RNA chaperone Hfq (*ssr3341*) is found to be conserved upstream of ComFB homologs in the unicellular cyanobacterial species, while it seems absent from the multicellular filamentous cyanobacteria of order *Nostocales*. Hfq protein is essential for phototaxis and natural competence, which depends on type IV pili (Dienst et al. 2008). Hfq regulates these processes by binding to the PilB1 ATPase subunit of pili machinery (Schuergers et al. 2014). Another conserved ORF found in association with *comFB* is CikA (*slr1969;* circadian input kinase A), encoding for a photoreceptor regulator of the circadian clock in cyanobacteria (Cohen & Golden 2015; Narikawa et al. 2008). The sigma factors are also involved in circadian clock or pili machinery regulation (Nair et al. 2002; Suban et al. 2024). Since ComFB is found in genomic organization with Hfq and circadian clock components, it seems logical that these proteins are also related in their function. Therefore, this further implies the involvement of ComFB in the regulation of light-dependent processes like natural competence or phototaxis (Taton et al. 2020; Menon et al. 2021), which are type IV pili dependent processes.

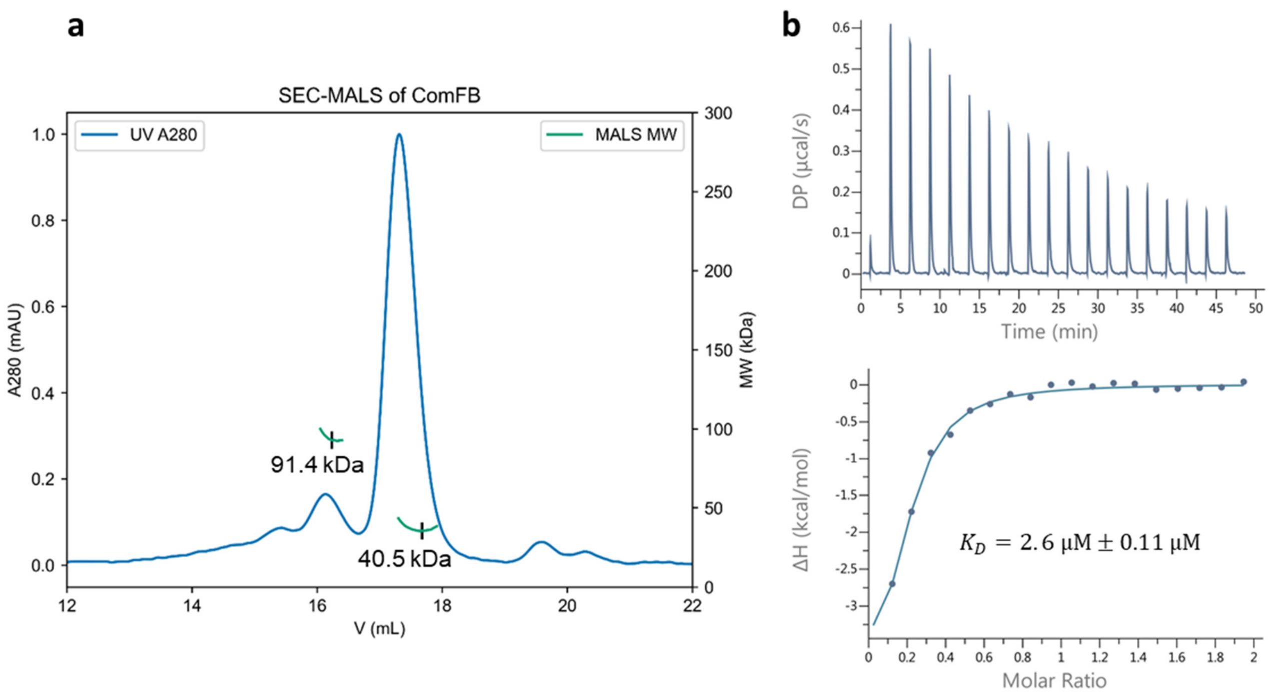

Supplementary Fig. S6. Characterization of ComFB protein encoded by *slr1970*. (a) Size exclusion chromatography coupled to multiangle light scattering (SEC-MALS) of recombinantly purified ComFB. Absorption at 280 nm (A280) is plotted against elution volume (V) from a Superose 6 Increase 10/300 GL column. Molecular weight (MW) obtained from MALS is plotted for the two main peaks in the A280 signal, with minima in the MW marked. The major peak of ComFB (thermotical mass of monomer 20.6 kDa) showed a ~ 40.5 kDa molar mass, indicating that ComFB behaves as a dimmer in solution, however a small fraction of the protein behaved as a tetramer as indicated by 91. kDa molar mass. **(b)** Representative isothermal titration calorimetry (ITC) measurements of 172 µM ComFB (monomeric concentration) titrated with 1 mM c-di-AMP. Upper panel shows the recorded differential power (DP) signal of ligand-to-protein titration, plotted against time. The enthalpy changes for each injected (ΔH) are calculated by subtraction of a differential power signal from buffer-in-protein titration control, and subsequent integration of the DP peaks, and plotted against molar ratio of ligand to protein. Lower panel shows the binding isotherms and the best-fit curves according to the one-set of binding sites for dimeric ComFB with K_D of 2.6 ± 0.11 µM.

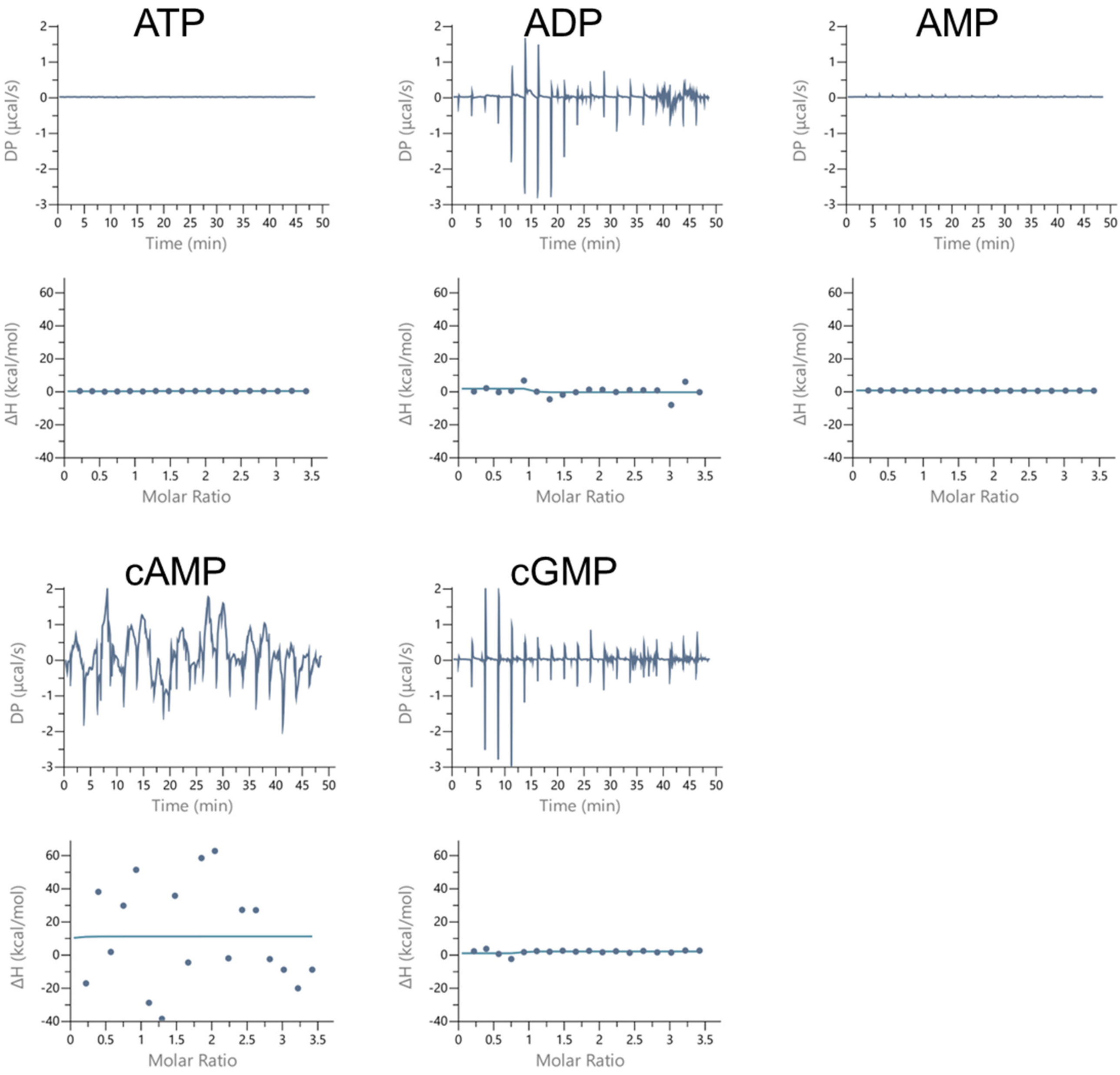

Supplementary Fig. S7. Binding analysis of ComFB to different nucleotides. Representative isothermal titration calorimetry (ITC) measurements of 114 µM ComFB (monomeric concentration) titrated with 1 mM of different nucleotides, as indicated. The Upper panel shows the recorded differential power (DP) signal of ligand-to-protein titration, plotted against time. The lower panel shows the binding isotherms and the fit curves according to the one-set of binding sites for ComFB. All of the tested ligands showed no binding to ComFB, confirming the specificity of c-di-NMP binding to ComFB.

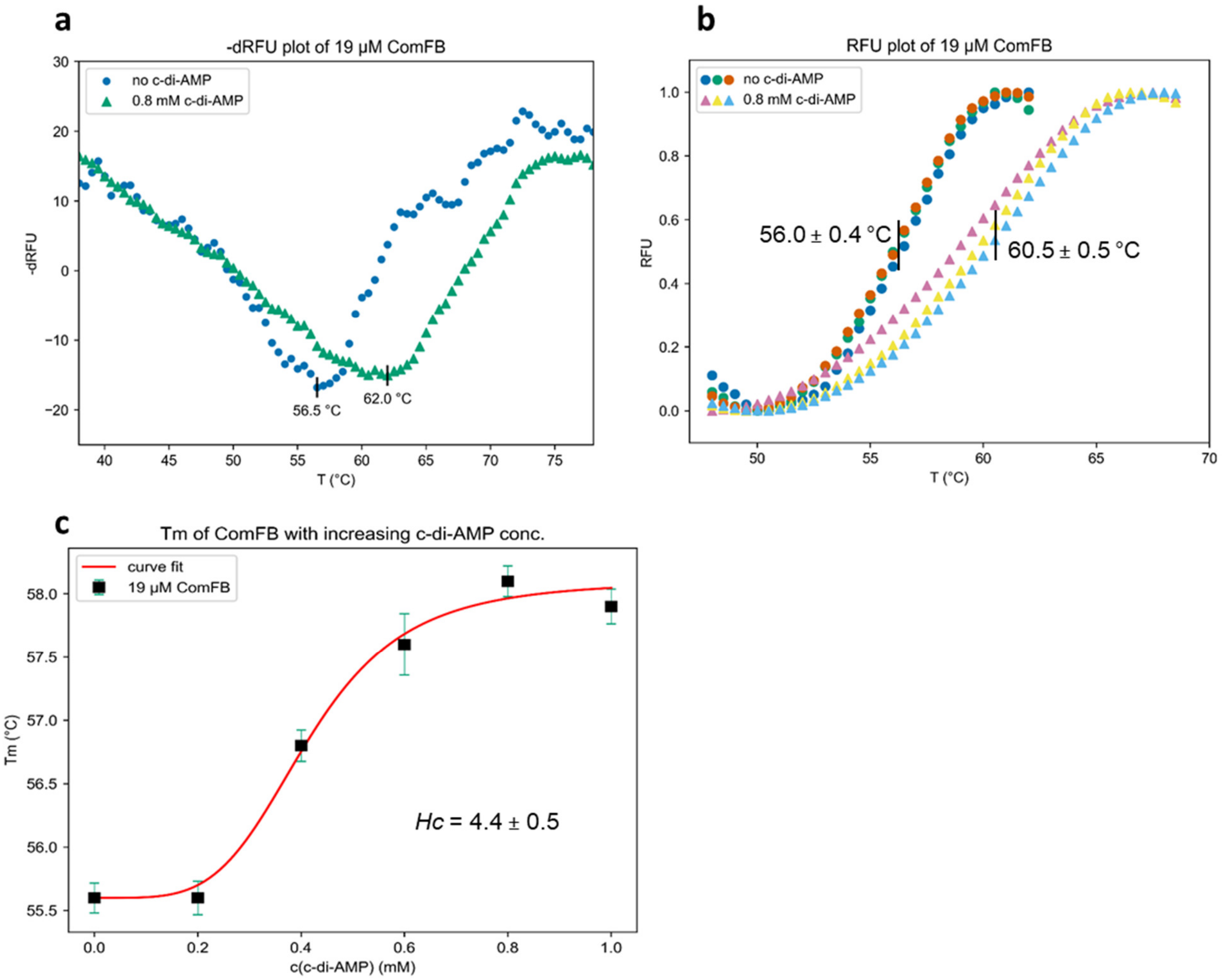

Supplementary Fig. S8. Thermal shift assay showing binding of c-di-AMP to ComFB. (a) Negative first derivative of representative ComFB (19 μM) melting profiles with and without c-di-AMP (0.8 mM), calculated from thermal shift assay data. Minima in the calculated negative first derivative of recorded relative fluorescence units (-dRFU) over temperature (T) represent the melting temperatures of the protein. **(b)** Melting profile of 19 μM ComFB with and without 0.8 mM c-di-AMP, recorded in a thermal shift assay. The fluorescence emission of SYPRO Orange at 570 nm was followed over a temperature range of 25-99 ℃, and normalised relative fluorescence units (RFU) were plotted against temperature (T). Temperatures at half-maximal normalised fluorescence emission are indicated. Measurements were performed in triplicates. **(c)** Melting temperatures (Tm) of 19 μM ComFB in the presence of different concentrations of c-di-AMP; Melting temperatures were calculated from -dRFU/dT plots as in **(a)**. Measurements were performed in triplicates; error bars show the standard deviation from the calculated mean melting temperature. The data were fitted with the Hill equation. Hill coefficient (*Hc*) is in positive value, indicative of cooperativity between binding sites. Calculated values of the fitting parameters and their variance are indicated.

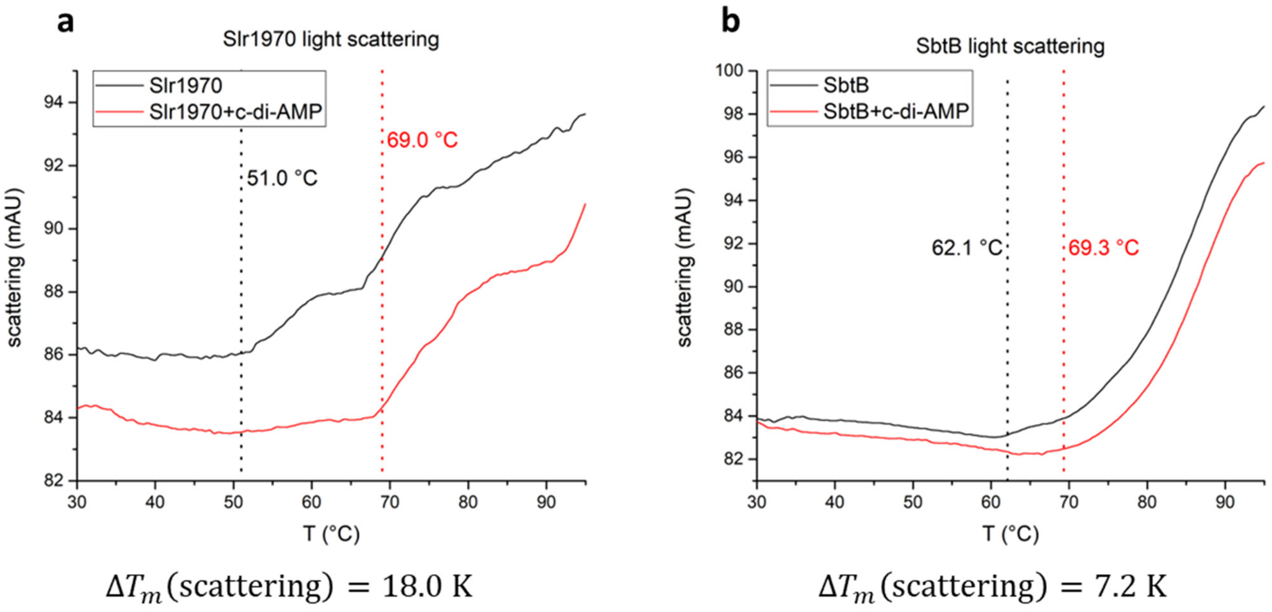

Supplementary Fig. S9. Light scattering obtained from the thermal shift assay using nanoDSF. The calculated temperatures from which the light scattering increases are shown as indicated. The temperature shift (ΔT_m) between proteins (1.5 mg/ml) with and without c-di-AMP (0.5 mM) is shown as indicated. **(a)** Light scattering of ComFB (Slr1970), while **(b)** light scattering of SbtB (used as +ve control as known c-di-AMP receptor protein) (Selim et al. 2021a).

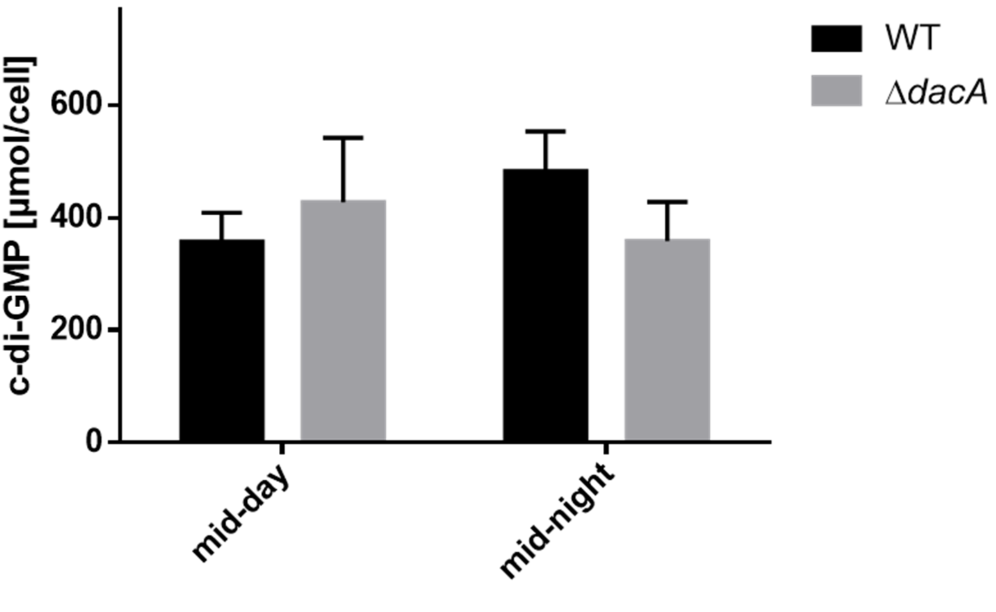

Supplementary Fig. S10. c-di-GMP concentration throughout a 12 h diurnal rhythm within *Synechocystis* WT (black bars) and ∆dacA cells (gray bars) at either mid of day or night phase (i.e. 6 h of light or darkness). X-axis shows the time in hours; Y-axis shows the intracellular concentrations of c-di-GMP.

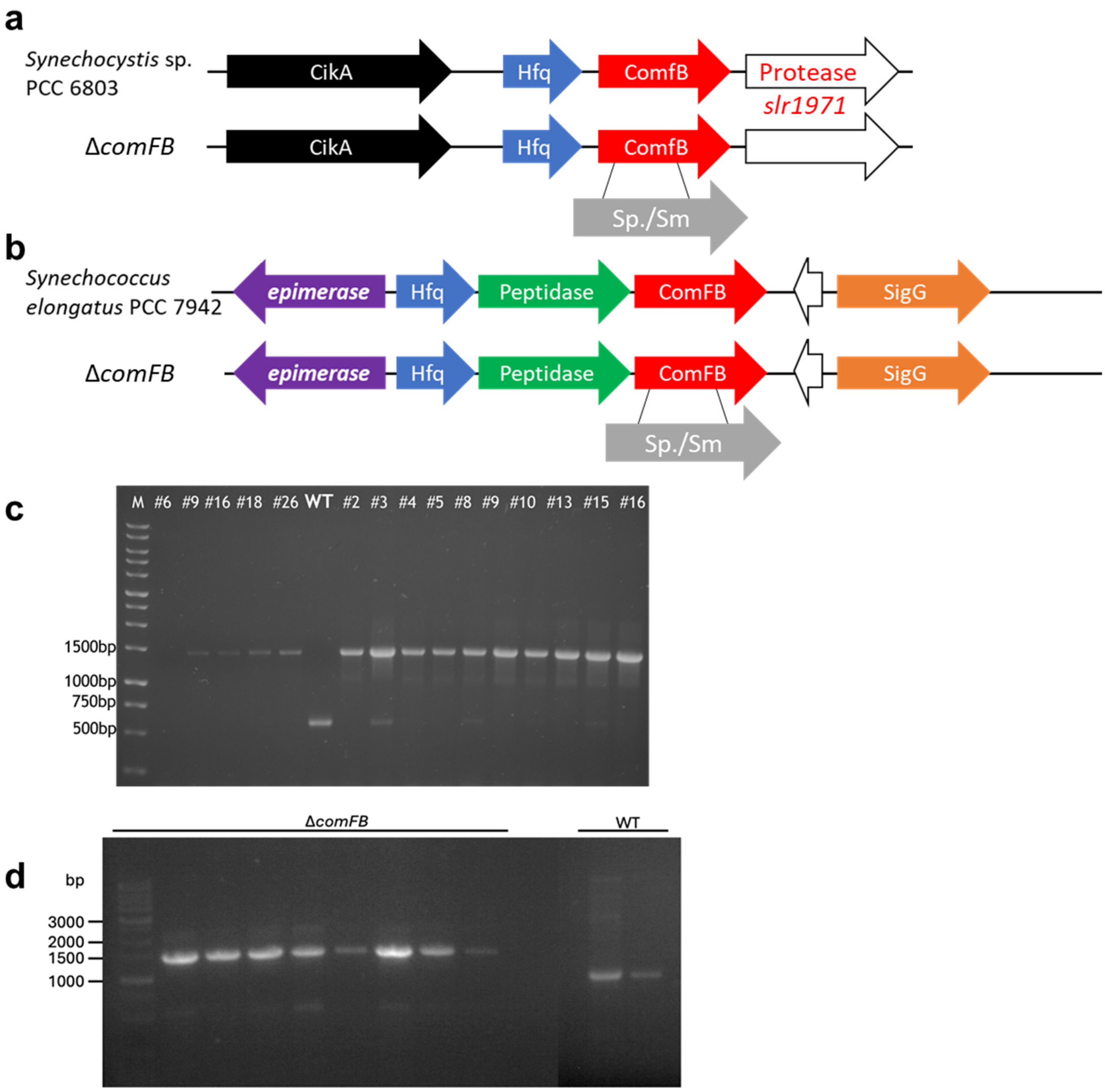

Supplementary Fig. S11. Genotypic characterization of Δ*comFB* knockout mutant in *Synechocystis* sp. PCC 6803 and *Synechococcus elongatus* PCC 7942. (a,b) Schematic representation of genetic organization of *slr1970* and *Synpcc7942_1924* (designated *comFB*) genes in *Synechocystis* sp. PCC 6803 **(a)** and *S. elongatus* PCC 7942 **(b)** genomes, respectively. The deletion of the *comFB* gene is achieved by a replacement with spectinomycin/streptomycin (Sp./Sm.) resistance cassette. **(c,d)** PCR showing complete segregation of Sp./Sm-resistance-cassette in the Δ*comFB* knockout mutants in *Synechocystis* **(c)** and *S. elongatus* **(d)** from independent colonies. The PCR product for the Δ*comF*B knockout mutants are around 1500 bp and appears higher than the corresponding WT band, indicating the insertion of Sp./Sm. resistance cassette.

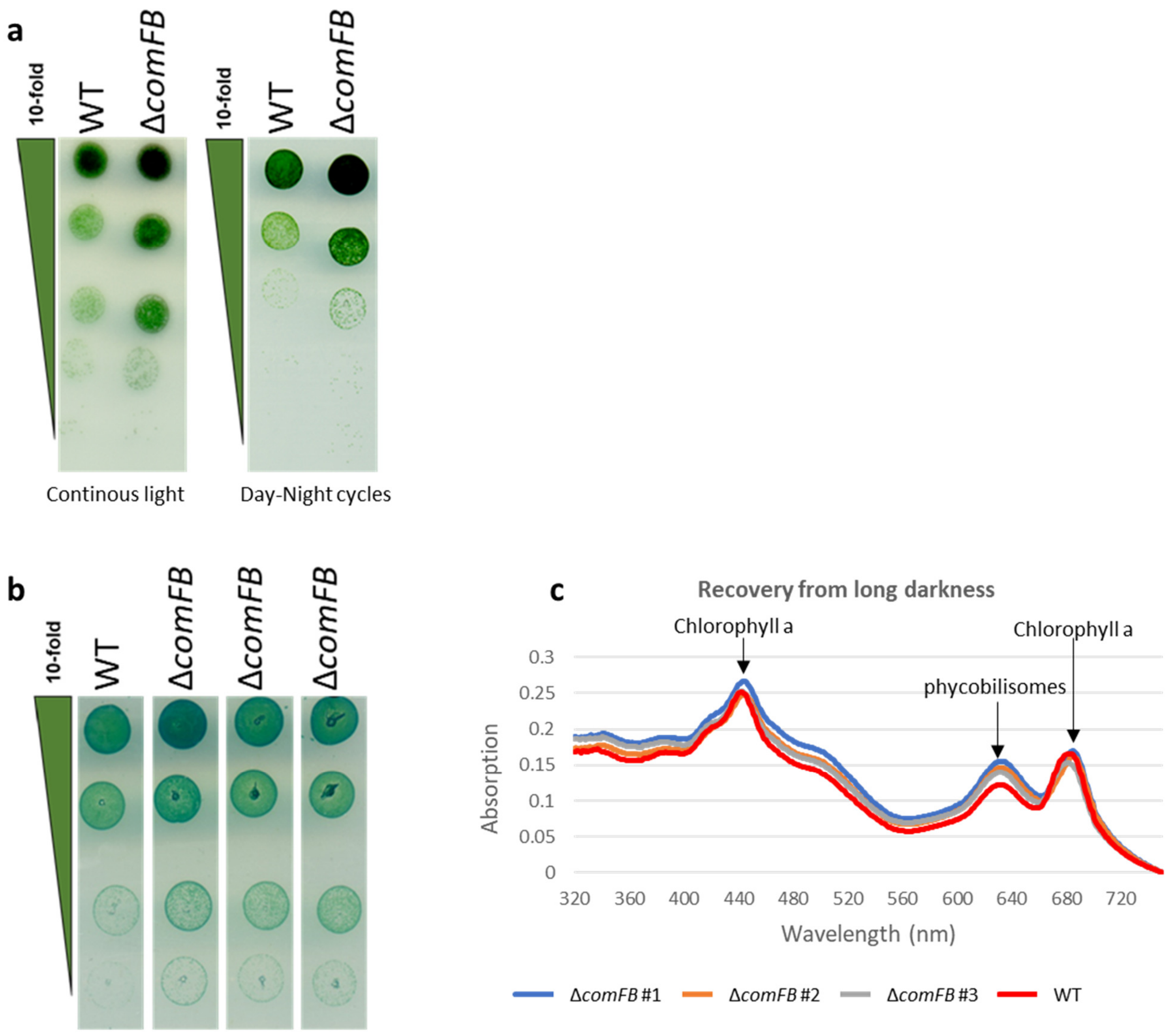

Supplementary Fig. S12. Phenotypic characterization of Δ*comFB* under different light conditions. (a) Growth test by drop plate assay of *Synechocystis* WT and Δ*comFB* cells under either continuous light (left) or a 12-hour diurnal rhythm (right). **(b)** Viability test using the drop-plate assay of *Synechocystis* WT and Δ*comFB* (3 independent clones of the mutant) cells after 6 days of incubation in complete darkness. Cells were normalized to an optical density at 750 nm (OD_{750}) of 1.0 and serial diluted in 10-fold steps (top to bottom; depicted by a green triangle). **(c)** Whole cell spectra of *Synechocystis* WT cells in comparison to Δ*comFB* cells after 2 days of recovery from darkness (6 days). The peak representing phycobilisomes as well as the peaks representing chlorophyll a are depicted by black arrows. Cultures were normalized to similar OD_{750}.

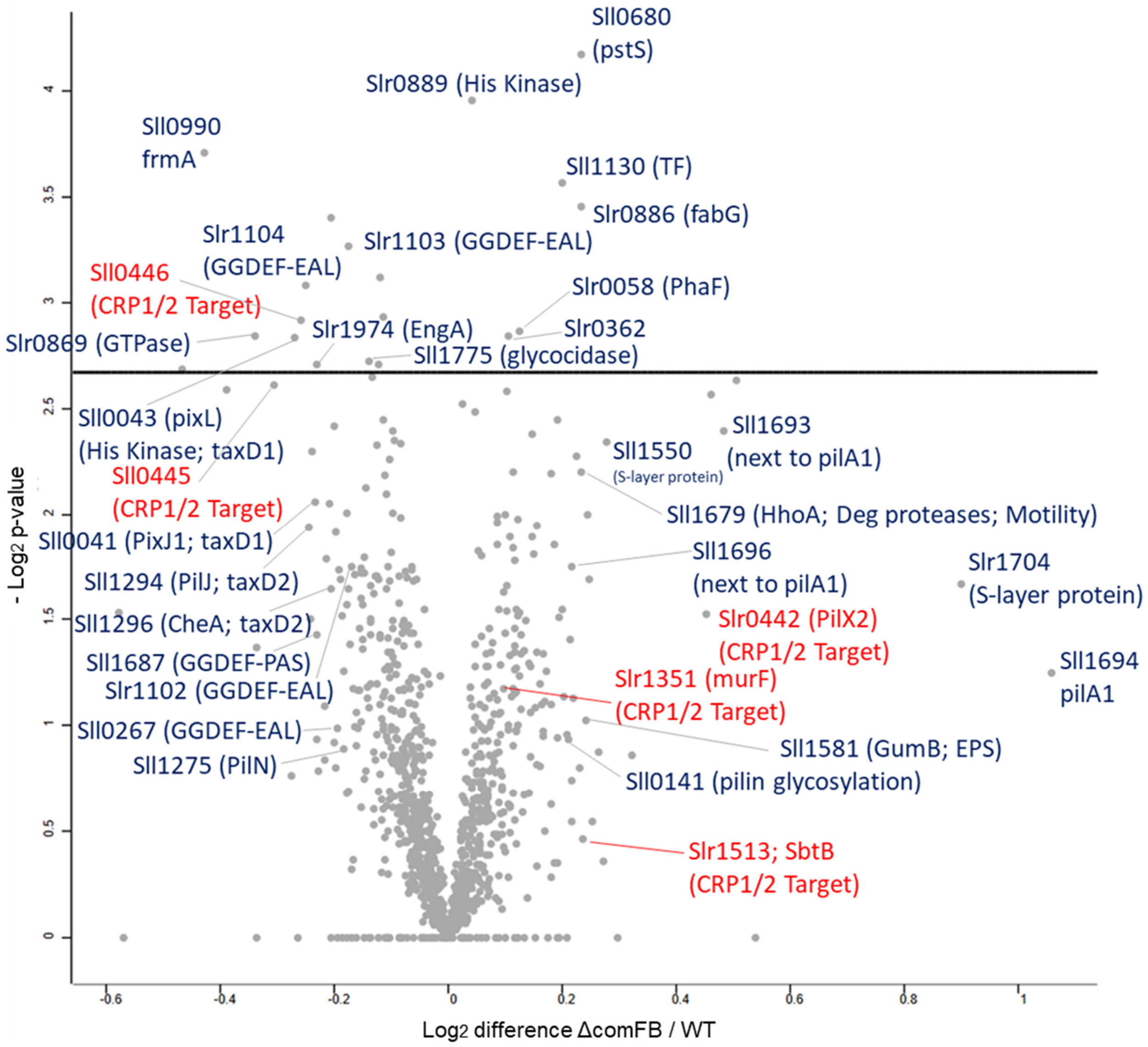

Supplementary Fig. S13. Proteome alterations of Δ*comFB* mutant compared to *Synechocystis* wildtype (WT) cells. Quantitative comparison of protein abundance between Δ*comFB* and WT with corresponding volcano plots indicate differences in protein abundance (Log_2 difference Δ*comFB*/WT) and corresponding $-log_{10}$ p-values from *t*-test of three independent replicates per strain. Proteins with significant changes in abundance are labeled with positive or negative values, indicating upregulation or downregulation in Δ*comFB* mutant, respectively.

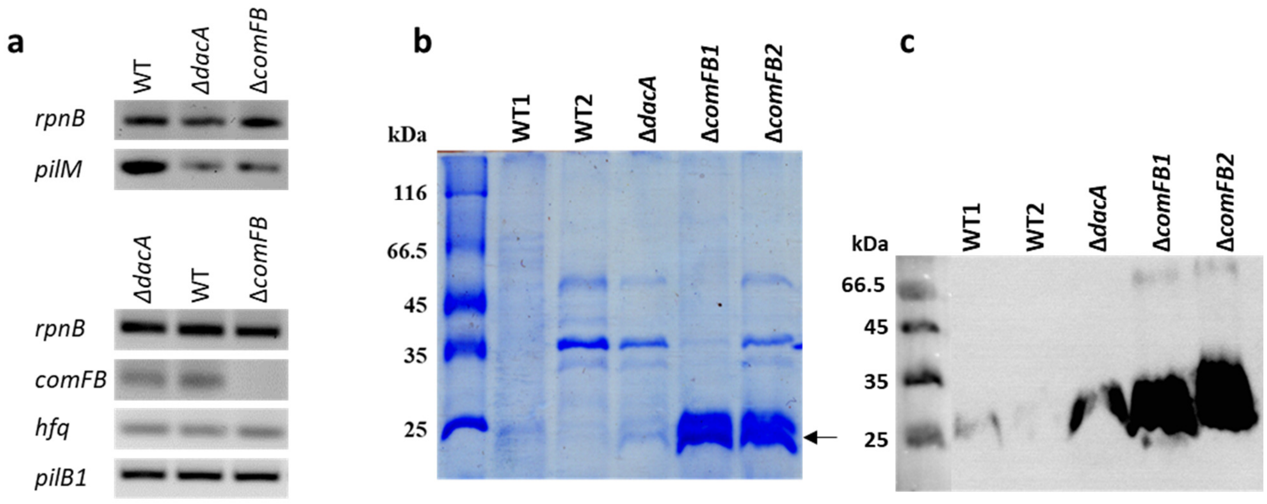

Supplementary Fig. S14. Characterization of *Synechocystis* Δ*dacA* and Δ*comFB* mutants. (a) Gene expression of selected pilus machinery genes in *Synechocystis* wildtype (WT) and Δ*dacA* and Δ*comFB* mutants analyzed using semiquantitative RT-PCR. The constitutively expressed *rnpB* gene served as loading control; a representative of 2 independent biological replicates is shown. **(b)** Exoproteome of WT, Δ*dacA* and Δ*comFB* cells, separated on SDS-PAGE and stained by coomassie blue and showing accumulation of PilA1 (indicated by black arrow). **(c)** Immunodetection of PilA1 in the exoproteome of WT and Δ*comFB* mutant for 2 independent biological replicates (indicated by 1 and 2). The Δ*dacA* mutant was used as positive control.

Co-fitness data for *comFB* mutants			
**Hit/Genes**	**Name**	**Description**	**Co-fitness**
Synpcc7942_0600	prkE (spkA)	Serine/threonine protein kinase	0.98
Synpcc7942_1110	cheY-like	response regulator receiver domain protein (CheY-like) involved in motility	0.93
Synpcc7942_2484	pilA	a candidate for PilA protein	0.92
Synpcc7942_1281		hypothetical protein	0.92
Synpcc7942_1926	hfq	hypothetical protein	0.91
Synpcc7942_0168		hypothetical Lipoprotein protein	0.91
Synpcc7942_2479	pilA2	Pilin-like protein	0.91
Synpcc7942_1436		hypothetical protein (frequently associate with IV pilus machinery)	0.91
Synpcc7942_2485	rntB (pilA candidate)	a candidate for PilA protein, required for natural transformation	0.90
Synpcc7942_1139	hmpF	Homologe of N. *punctiforme* HmpF motility protein	0.90
Synpcc7942_2450	pilQ	General secretion pathway protein D	0.90
Synpcc7942_2486	rntA	required for natural transformation	0.90
Synpcc7942_2071	pilB1	ATPase	0.90
Synpcc7942_0862	ebsA	Pilus machinery protein EbsA (essential for biofilm suppression protein A)	0.90
Synpcc7942_2451	pilO	IV pilus assembly protein PilO	0.90
Synpcc7942_2453	pilM	type IV pilus assembly protein PilM	0.90
Synpcc7942_2452	pilN	type IV pilus assembly protein PilN-like	0.90
Synpcc7942_2069	pilC	fimbrial assembly protein PilC-like	0.90
Synpcc7942_1510	sigF1	RNA polymerase sigma factor SigF	0.89
Synpcc7942_0049	pilA	pilin polypeptide PilA-like	0.85

Supplementary Fig. S15. Co-fitness data for *Synechococcus elongatus* Δ*comFB* mutant within RB-TnSeq (Random Barcode Transposon Insertion Site Sequencing) library conditions (Wetmore et al. 2015; Price et al. 2018).

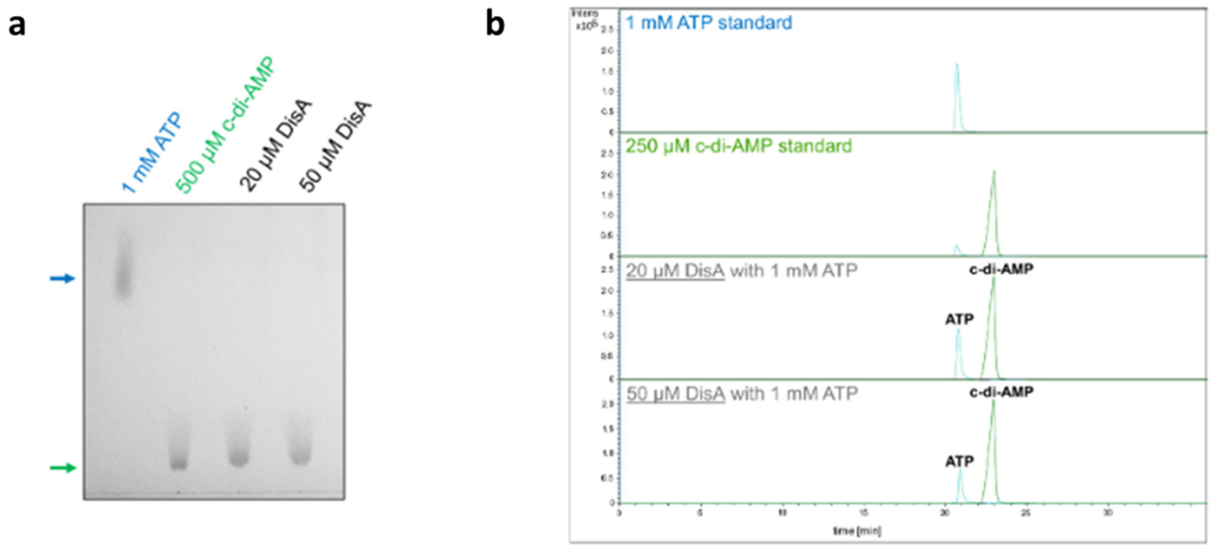

Supplementary Fig. S16. Synthesis of c-di-AMP from purified DisA. Analysis of the enzymatic conversion of 1 mM ATP to c-di-AMP with 20 µM or 50 µM recombinant DisA. (a) Thin layer chromatography (TLC). Arrows denote running distance of c-di-AMP (green) and ATP (blue). **(b)** LC-MS analysis of purified His_6-tagged DisA showing conversion of ATP to c-di-AMP.

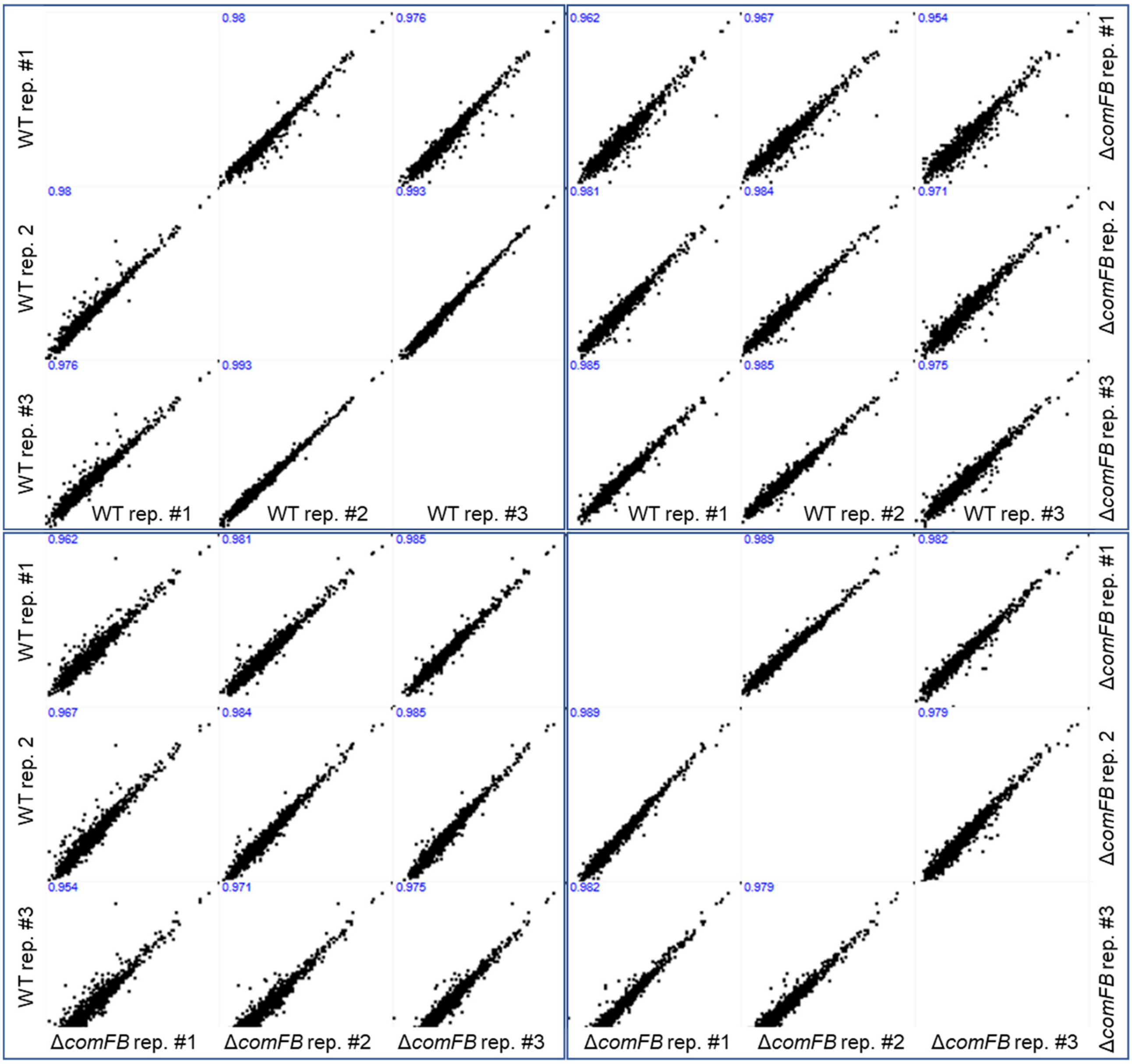

Supplementary Fig. S17. Intensity-based correlation of replicated proteome analyses. Shown is the correlation of protein intensities (in Log_{10} scale) between independent replicates the wildtype (WT) and Δ*comFB* strains. Pearson correlation coefficients are indicated for correlations between each two replicates within the strain and between different strains.

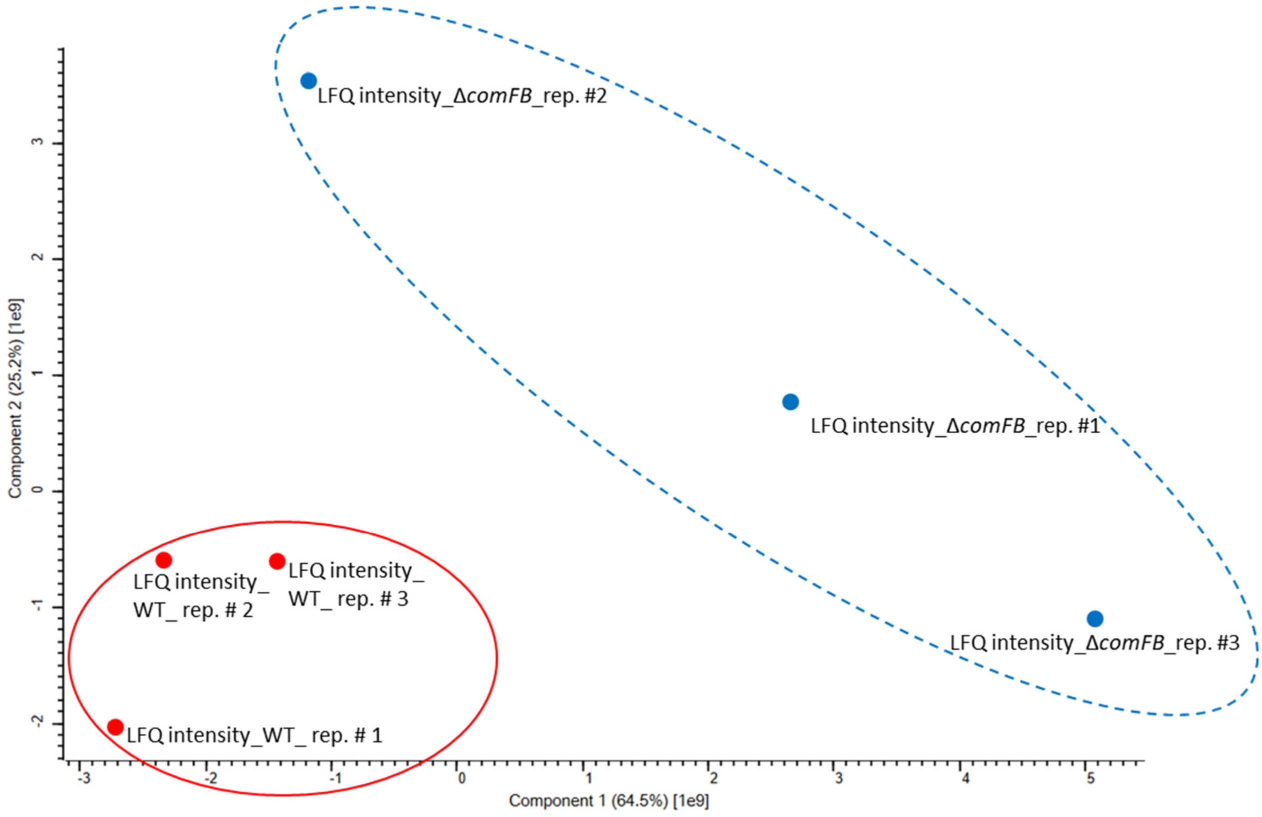

Supplementary Fig. S18. Proteomic landscape of Δ*comFB* mutant in comparison to *Synechocystis* wildtype (WT) cells. Principal component analysis of protein abundance in the wildtype (red circles) and Δ*comFB* (blue circles) is shown. Co-clustering of wildtype replicates indicates similar proteome compositions. Offset clustering of Δ*comFB* (dotted ellipse) indicates broad changes in protein abundance compared to wildtype.

Supporting Tables (S1 to S5)

Legend for Supporting Tables S1 to S5

Supplemental Table **S1**	List of identified proteins involved in motility and pilus biogenesis and corresponding LFQ intensities in dacA (sll0505) mutant compared to WT cells
Supplemental Table **S2**	List of Protein-coding genes involved in motility and pilus biogenesis in dacA (sll0505) mutant compared to WT cells
Supplemental Table **S3**	List of identified proteins involved in motility and pilius biogenesis and corresponding LFQ intensities in sbtB (slr1513) mutant compared to WT cells
Supplemental Table **S4**	List of identified proteins and corresponding LFQ intensities in comFB (slr1970) mutant compared to WT cells
Supplemental Table **S5**	Primers used in this study to generate complementation plasmids

Tables S1 to S5 are in separate Excel files.

Supplementary References

- Bhaya D, Bianco NR, Bryant D, Grossman A. Type IV pilus biogenesis and motility in the cyanobacterium Synechocystis sp. PCC6803. Mol Microbiol. 2000; 37(4):941-51. doi: 10.1046/j.1365-2958.2000.02068.x.
- Cengic I, Uhlén M, Hudson EP. Surface Display of Small Affinity Proteins on Synechocystis sp. Strain PCC 6803 Mediated by Fusion to the Major Type IV Pilin PilA1. J Bacteriol. 2018; 200(16):e00270-18. doi: 10.1128/JB.00270-18.
- Chang YW, Rettberg LA, Treuner-Lange A, Iwasa J, Søgaard-Andersen L, Jensen GJ. Architecture of the type IVa pilus machine. Science. 2016 Mar 11;351(6278):aad2001. doi: 10.1126/science.aad2001. Epub 2016 Mar 10. Erratum in: Science. 2016; 352(6282). pii: aaf7977. doi: 10.1126/science.aaf7977.
- Chen Z, Li X, Tan X, Zhang Y, Wang B. Recent Advances in Biological Functions of Thick Pili in the Cyanobacterium *Synechocystis* sp. PCC 6803. Front Plant Sci. 2020; 11:241. doi: 10.3389/fpls.2020.00241.
- Cohen SE, Golden SS. Circadian Rhythms in Cyanobacteria. Microbiol Mol Biol Rev. 2015; 79(4):373-85. doi: 10.1128/MMBR.00036-15.
- Conradi FD, Mullineaux CW, Wilde A. The Role of the Cyanobacterial Type IV Pilus Machinery in Finding and Maintaining a Favourable Environment. Life (Basel). 2020; 10(11):252. doi: 10.3390/life10110252.
- Dienst D, Dühring U, Mollenkopf HJ, Vogel J, Golecki J, Hess WR, Wilde A. The cyanobacterial homologue of the RNA chaperone Hfq is essential for motility of Synechocystis sp. PCC 6803. Microbiology (Reading). 2008; 154(Pt 10):3134-3143. doi: 10.1099/mic.0.2008/020222-0.
- Ellison CK, Dalia TN, Vidal Ceballos A, Wang JC, Biais N, Brun YV, Dalia AB. Retraction of DNA-bound type IV competence pili initiates DNA uptake during natural transformation in Vibrio cholerae. Nat Microbiol. 2018; 3(7):773-780. doi: 10.1038/s41564-018-0174-y.
- Forchhammer K, Selim KA, Huergo LF. New views on PII signaling: from nitrogen sensing to global metabolic control. Trends Microbiol. 2022; 30(8):722-735. doi: 10.1016/j.tim.2021.12.014.
- Foster AJ, van den Noort M, Poolman B. Bacterial cell volume regulation and the importance of cyclic di-AMP. Microbiol Mol Biol Rev. 2024; 88(2):e0018123. doi: 10.1128/mmbr.00181-23.
- Gibson PS, Veening JW. Gaps in the wall: understanding cell wall biology to tackle amoxicillin resistance in Streptococcus pneumoniae. Curr Opin Microbiol. 2023; 72:102261. doi: 10.1016/j.mib.2022.102261.
- Gonçalves CF, Pacheco CC, Tamagnini P, Oliveira P. Identification of inner membrane translocase components of TolC-mediated secretion in the cyanobacterium Synechocystis sp. PCC 6803. Environ Microbiol. 2018; 20(7):2354-2369. doi: 10.1111/1462-2920.14095.
- Haffner M, Hou W-T, Mantovani O, Walke PR, Hauf K, Borisova M, Hagemann M, Zhou C-Z, Forchhammer K, Selim KA. PII signal transduction superfamily acts as a valve plug to control bicarbonate and ammonia homeostasis among different bacterial phyla. BioRxiv 2023a; doi: https://doi.org/10.1101/2023.08.10.552651

- Haffner M, Mantovani O, Spät P, Maček B, Hagemann M, Forchhammer K, Selim KA. Diurnal rhythm causes metabolic crises in the cyanobacterial mutants of c-di-AMP signalling cascade. BioRxiv 2023b; doi: https://doi.org/10.1101/2023.11.14.567006.
- Haft DH, Badretdin A, Coulouris G, DiCuccio M, Durkin AS, Jovenitti E, Li W, Mersha M, O'Neill KR, Virothaisakun J, Thibaud-Nissen F. RefSeq and the prokaryotic genome annotation pipeline in the age of metagenomes. Nucleic Acids Res. 2023; gkad988. doi: 10.1093/nar/gkad988.
- He J, Yin W, Galperin MY, Chou SH. Cyclic di-AMP, a second messenger of primary importance: tertiary structures and binding mechanisms. Nucleic Acids Res. 2020; 48(6):2807-2829. doi: 10.1093/nar/gkaa112.
- Herzberg C, Meißner J, Warneke R, Stülke J. The many roles of cyclic di-AMP to control the physiology of *Bacillus subtilis*. Microlife. 2023; 4:uqad043. doi: 10.1093/femsml/uqad043.
- Krol E, Werel L, Essen LO, Becker A. Structural and functional diversity of bacterial cyclic nucleotide perception by CRP proteins. Microlife. 2023; 4:uqad024. doi: 10.1093/femsml/uqad024.
- Kronborg K, Zhang YE. cAMP competitively inhibits periplasmic phosphatases to coordinate nutritional growth with competence of *Haemophilus influenzae*. J Biol Chem. 2023; 105404. doi: https://doi.org/10.1016/j.jbc.2023.105404
- Lapina T, Selim KA, Forchhammer K, Ermilova E. The PII signaling protein from red algae represents an evolutionary link between cyanobacterial and Chloroplastida PII proteins. Sci Rep. 2018; 8(1):790. doi: 10.1038/s41598-017-19046-7.
- Letunic I, Bork P. Interactive Tree Of Life (iTOL) v5: an online tool for phylogenetic tree display and annotation. Nucleic Acids Res. 2021; 49(W1):W293-W296. doi: 10.1093/nar/gkab301.
- Linhartová M, Bučinská L, Halada P, Ječmen T, Setlík J, Komenda J, Sobotka R. Accumulation of the Type IV prepilin triggers degradation of SecY and YidC and inhibits synthesis of Photosystem II proteins in the cyanobacterium Synechocystis PCC 6803. Mol Microbiol. 2014; 93(6):1207-23. doi: 10.1111/mmi.12730.
- Linhartová M, Skotnicová P, Hakkila K, Tichý M, Komenda J, Knoppová J, Gilabert JF, Guallar V, Tyystjärvi T, Sobotka R. Mutations Suppressing the Lack of Prepilin Peptidase Provide Insights into the Maturation of the Major Pilin Protein in Cyanobacteria. Front Microbiol. 2021; 12:756912. doi: 10.3389/fmicb.2021.756912.
- Mantovani O, Haffner M, Selim KA, Hagemann M, Forchhammer K. Roles of second messengers in the regulation of cyanobacterial physiology: the carbon-concentrating mechanism and beyond. Microlife. 2023a; 4:uqad008. doi: 10.1093/femsml/uqad008.
- Mantovani O, Haffner M, Walke P, Elshereef AA, Wagner B, Petras D, Forchhammer K, Selim KA, Hagemann M. The redox-sensitive R-loop of the carbon control protein SbtB contributes to the regulation of the cyanobacterial CCM. Scientific Reports 2024; 14: 7885.
- Mantovani O, Reimann V, Haffner M, Herrmann FP, Selim KA, Forchhammer K, Hess WR, Hagemann M. The impact of the cyanobacterial carbon-regulator protein SbtB and of the second messengers cAMP and c-di-AMP on CO_2 -dependent gene expression. New Phytol. 2022; 234(5):1801-1816. doi: 10.1111/nph.18094.

- Mehne FM, Gunka K, Eilers H, Herzberg C, Kaever V, Stülke J. Cyclic di-AMP homeostasis in bacillus subtilis: both lack and high level accumulation of the nucleotide are detrimental for cell growth. J Biol Chem. 2013; 288(3):2004-17. doi: 10.1074/jbc.M112.395491.
- Menon SN, Varuni P, Bunbury F, Bhaya D, Menon GI. Phototaxis in Cyanobacteria: From Mutants to Models of Collective Behavior. mBio. 2021; 12(6):e0239821. doi: 10.1128/mBio.02398-21.
- Mistry J, Chuguransky S, Williams L, Qureshi M, Salazar GA, Sonnhammer ELL, Tosatto SCE, Paladin L, Raj S, Richardson LJ, Finn RD, Bateman A. Pfam: The protein families database in 2021. Nucleic Acids Res. 2021; 49(D1):D412-D419. doi: 10.1093/nar/gkaa913.
- Nair U, Ditty JL, Min H, Golden SS. Roles for sigma factors in global circadian regulation of the cyanobacterial genome. J Bacteriol. 2002; 184(13):3530-8. doi: 10.1128/JB.184.13.3530-3538.2002.
- Narikawa R, Kohchi T, Ikeuchi M. Characterization of the photoactive GAF domain of the CikA homolog (SyCikA, Slr1969) of the cyanobacterium Synechocystis sp. PCC 6803. Photochem Photobiol Sci. 2008; 7(10):1253-9. doi: 10.1039/b811214b.
- Nelson JW, Sudarsan N, Furukawa K, Weinberg Z, Wang JX, Breaker RR. Riboswitches in eubacteria sense the second messenger c-di-AMP. Nat Chem Biol. 2013; 9(12):834-9. doi: 10.1038/nchembio.1363.
- Neumann N, Friz S, Forchhammer K. Glucose-1,6-Bisphosphate, a Key Metabolic Regulator, Is Synthesized by a Distinct Family of α-Phosphohexomutases Widely Distributed in Prokaryotes. mBio. 2022; 13(4):e0146922. doi: 10.1128/mbio.01469-22.
- Oeser S, Wallner T, Schuergers N, Bučinská L, Sivabalasarma S, Bähre H, Albers SV, Wilde A. Minor pilins are involved in motility and natural competence in the cyanobacterium Synechocystis sp. PCC 6803. Mol Microbiol. 2021 Sep;116(3):743-765. doi: 10.1111/mmi.14768.
- Okamoto S, Ohmori M. The cyanobacterial PilT protein responsible for cell motility and transformation hydrolyzes ATP. Plant Cell Physiol. 2002; 43(10):1127-36. doi: 10.1093/pcp/pcf128.
- Price MN, Wetmore KM, Waters RJ, Callaghan M, Ray J, Liu H, Kuehl JV, Melnyk RA, Lamson JS, Suh Y, Carlson HK, Esquivel Z, Sadeeshkumar H, Chakraborty R, Zane GM, Rubin BE, Wall JD, Visel A, Bristow J, Blow MJ, Arkin AP, Deutschbauer AM. Mutant phenotypes for thousands of bacterial genes of unknown function. Nature. 2018; 557(7706):503-509. doi: 10.1038/s41586-018-0124-0.
- Ren A, Patel DJ. c-di-AMP binds the ydaO riboswitch in two pseudo-symmetry-related pockets. Nat Chem Biol. 2014; 10(9):780-6. doi: 10.1038/nchembio.1606.
- Roelofs KG, Wang J, Sintim HO, Lee VT. Differential radial capillary action of ligand assay for high-throughput detection of protein-metabolite interactions. Proc Natl Acad Sci USA. 2011; 108(37):15528-33. doi: 10.1073/pnas.1018949108.
- Rubin BE, Huynh TN, Welkie DG, Diamond S, Simkovsky R, Pierce EC, Taton A, Lowe LC, Lee JJ, Rifkin SA, Woodward JJ, Golden SS. High-throughput interaction screens illuminate the role of c-di-AMP in cyanobacterial nighttime survival. PLoS Genet. 2018; 14(4):e1007301. doi: 10.1371/journal.pgen.1007301.
- Samir S, Elshereef AA, Alva V, Hahn J, Dubnau D, Galperin MY, Selim KA. ComFB, a new widespread family of c-di-NMP receptor proteins. BioRxiv. 2024; doi: 10.1101/2024.11.10.622515.

- Schirmacher AM, Hanamghar SS, Zedler JAZ. Function and Benefits of Natural Competence in Cyanobacteria: From Ecology to Targeted Manipulation. Life (Basel). 2020; 10(11):249. doi: 10.3390/life10110249.
- Schuergers N, Ruppert U, Watanabe S, Nürnberg DJ, Lochnit G, Dienst D, Mullineaux CW, Wilde A. Binding of the RNA chaperone Hfq to the type IV pilus base is crucial for its function in Synechocystis sp. PCC 6803. Mol Microbiol. 2014; 92(4):840-52. doi: 10.1111/mmi.12595.
- Schumacher MA, Lent N, Chen VB, Salinas R. Structures of the DarR transcription regulator reveal unique modes of second messenger and DNA binding. Nat Commun. 2023; 14(1):7239. doi: 10.1038/s41467-023-42823-0.
- Selim KA, Alva V. PII-like signaling proteins: a new paradigm in orchestrating cellular homeostasis. Curr Opin Microbiol. 2024; 79:102453. doi: 10.1016/j.mib.2024.102453.
- Selim KA, Haase F, Hartmann MD, Hagemann M, Forchhammer K. P_{II}-like signaling protein SbtB links cAMP sensing with cyanobacterial inorganic carbon response. Proc Natl Acad Sci USA. 2018; 115(21):E4861-E4869. doi: 10.1073/pnas.1803790115.
- Selim KA, Haffner M, Burkhardt M, Mantovani O, Neumann N, Albrecht R, Seifert R, Krüger L, Stülke J, Hartmann MD, Hagemann M, Forchhammer K. Diurnal metabolic control in cyanobacteria requires perception of second messenger signaling molecule c-di-AMP by the carbon control protein SbtB. Sci Adv. 2021a; 7(50):eabk0568. doi: 10.1126/sciadv.abk0568.
- Selim KA, Haffner M, Mantovani O, Albrecht R, Zhu H, Hagemann M, Forchhammer K, Hartmann MD. Carbon signaling protein SbtB possesses atypical redox-regulated apyrase activity to facilitate regulation of bicarbonate transporter SbtA. Proc Natl Acad Sci USA. 2023; 120(8):e2205882120. doi: 10.1073/pnas.2205882120.
- Selim KA, Haffner M, Watzer B, Forchhammer K. Tuning the in vitro sensing and signaling properties of cyanobacterial PII protein by mutation of key residues. Sci Rep. 2019; 9(1):18985. doi: 10.1038/s41598-019-55495-y.
- Selim KA, Lapina T, Forchhammer K, Ermilova E. Interaction of N-acetyl-l-glutamate kinase with the PII signal transducer in the non-photosynthetic alga Polytomella parva: Co-evolution towards a hetero-oligomeric enzyme. FEBS J. 2020; 287(3):465-482. doi: 10.1111/febs.14989.
- Selim KA, Tremiño L, Marco-Marín C, Alva V, Espinosa J, Contreras A, Hartmann MD, Forchhammer K, Rubio V. Functional and structural characterization of PII-like protein CutA does not support involvement in heavy metal tolerance and hints at a small-molecule carrying/signaling role. FEBS J. 2021b; 288(4):1142-1162. doi: 10.1111/febs.15464.
- Sergeyenko TV, Los DA. Identification of secreted proteins of the cyanobacterium *Synechocystis* sp. strain PCC 6803. FEMS Microbiol Lett. 2000; 193(2):213-6. doi: 10.1111/j.1574-6968.2000.tb09426.x.
- Shyp V, Dubey BN, Böhm R, Hartl J, Nesper J, Vorholt JA, Hiller S, Schirmer T, Jenal U. Reciprocal growth control by competitive binding of nucleotide second messengers to a metabolic switch in Caulobacter crescentus. Nat Microbiol. 2021; 6(1):59-72. doi: 10.1038/s41564-020-00809-4.
- Singh AK, Li H, Bono L, Sherman LA. Novel adaptive responses revealed by transcription profiling of a Synechocystis sp. PCC 6803 delta-isiA mutant in the presence and absence of hydrogen peroxide. Photosynth Res. 2005; 84(1-3):65-70. doi: 10.1007/s11120-004-6429-x.

- Stülke J, Krüger L. Cyclic di-AMP Signaling in Bacteria. Annu Rev Microbiol. 2020; 74:159-179. doi: 10.1146/annurev-micro-020518-115943.
- Suban S, Sendersky E, Golden SS, Schwarz R. Impairment of a cyanobacterial glycosyltransferase that modifies a pilin results in biofilm development. Environ Microbiol Rep. 2022; 14(2):218-229. doi: 10.1111/1758-2229.13050.
- Suban S, Yemini S, Shor A, Ben-Asher HW, Yaron O, Karako-Lampert S, Senderky E, Golden SS, Schwarz R. A cyanobacterial sigma factor F controls biofilm-promoting genes through intra- and intercellular pathways. Biofilm. 2024; 8: 2590-2075. doi: 10.1016/j.bioflm.2024.100217.
- Taton A, Erikson C, Yang Y, Rubin BE, Rifkin SA, Golden JW, Golden SS. The circadian clock and darkness control natural competence in cyanobacteria. Nat Commun. 2020; 11(1):1688. doi: 10.1038/s41467-020-15384-9.
- Walter J, Selim KA, Leganés F, Fernández-Piñas F, Vothknecht UC, Forchhammer K, Aro EM, Gollan PJ. A novel Ca^{2+}-binding protein influences photosynthetic electron transport in Anabaena sp. PCC 7120. Biochim Biophys Acta Bioenerg. 2019; 1860(6):519-532. doi: 10.1016/j.bbabio.2019.04.007.
- Werel L, Farmani N, Krol E, Serrania J, Essen LO, Becker A. Structural Basis of Dual Specificity of Sinorhizobium meliloti Clr, a cAMP and cGMP Receptor Protein. mBio. 2023; 14(2):e0302822. doi: 10.1128/mbio.03028-22.
- Wetmore KM, Price MN, Waters RJ, Lamson JS, He J, Hoover CA, Blow MJ, Bristow J, Butland G, Arkin AP, Deutschbauer A. Rapid Quantification of Mutant Fitness in Diverse Bacteria by Sequencing Randomly Bar-Coded Transposons. mBio. 2015; doi:10.1128/mbio.00306-15
- Yin W, Cai X, Ma H, Zhu L, Zhang Y, Chou SH, Galperin MY, He J. A decade of research on the second messenger c-di-AMP. FEMS Microbiol Rev. 2020; 44(6):701-724. doi: 10.1093/femsre/fuaa019.
- Yoon SH, Waters CM. The ever-expanding world of bacterial cyclic oligonucleotide second messengers. Curr Opin Microbiol. 2021; 60:96-103. doi: 10.1016/j.mib.2021.01.017.
- Yoshihara S, Geng X, Okamoto S, Yura K, Murata T, Go M, Ohmori M, Ikeuchi M. Mutational analysis of genes involved in pilus structure, motility and transformation competency in the unicellular motile cyanobacterium *Synechocystis* sp. PCC 6803. Plant Cell Physiol. 2001; 42(1):63-73. doi: 10.1093/pcp/pce007.
- Zarrella TM, Yang J, Metzger DW, Bai G. Bacterial Second Messenger Cyclic di-AMP Modulates the Competence State in Streptococcus pneumoniae. J Bacteriol. 2020; 202(4):e00691-19. doi: 10.1128/JB.00691-19.
- Zeng X, Huang M, Sun QX, Peng YJ, Xu X, Tang YB, Zhang JY, Yang Y, Zhang CC. A c-di-GMP binding effector controls cell size in a cyanobacterium. Proc Natl Acad Sci USA. 2023; 120(13):e2221874120. doi: 10.1073/pnas.2221874120.
